# Crystallographic Study of DNA T‐Junction via Crystal Engineering

**DOI:** 10.1002/anie.202518174

**Published:** 2025-10-30

**Authors:** Xiang Li, Naseem Siraj, Ruojie Sha, Chengde Mao

**Affiliations:** ^1^ Purdue University Department of Chemistry West Lafayette IN 47907 USA; ^2^ New York University Department of Chemistry New York NY 10003 USA

**Keywords:** DNA, DNA crystal engineering, DNA nanostructures, DNA nanotechnology

## Abstract

Engineering DNA crystals is the primary motivation for structural DNA nanotechnology. Among many potential applications, such crystals promise as a platform to precisely (in terms of both location and orientation) organize biomolecules into 3D crystals for X‐ray crystallographic studies of the guest biomolecules. The crystal formation depends on rationally designed DNA frameworks instead of unpredictable interactions between the guest molecule themselves; thus, avoiding the crystallization problem of biomolecules. This approach was proposed 40 years ago, however, has not been realized so far. Herein, we report an effort along this direction to study DNA T‐junction, a common DNA structure used in DNA nanoconstruction. This study is an initial demonstration of the feasibility of the 40‐year‐old proposal. We expect that it would be quickly adapted to study many other molecules, particularly nucleic acids (e.g., aptamers, catalytic DNAs/RNAs, ribozymes, and ribonucleoproteins) that are, otherwise, difficult to be studied.

## Graphical abstract

Engineered DNA crystals provides a platform to regularly arrange guest molecules for crystallographic study. Herein, the 3D structure of T‐junction, a commonly used motif in DNA nanotechnology, was revealed.

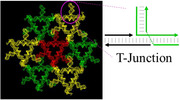

DNA nanotechnology^[^ ^1^, ^2^, ^3^, ^4^, ^5^, ^6^, ^7^, ^8^, ^9^ ^]^ including DNA crystal engineering^[^ ^10^, ^11^, ^12^, ^13^, ^14^, ^15^, ^16^, ^17^, ^18^, ^19^, ^20^, ^21^, ^22^, ^23^, ^24^, ^25^, ^26^, ^27^, ^28^, ^29^, ^30^, ^31^, ^32^, ^33^, ^34^, ^35^, ^36^, ^37^, ^38^, ^39^, ^40^, ^41^, ^42^ ^]^–as the original motivation for structural DNA nanotechnology–has rapidly evolved recently. Several general DNA motifs, including tensegrity triangles,^[^ ^10^, ^11^, ^12^, ^13^, ^14^, ^15^, ^16^, ^17^, ^18^, ^19^, ^20^, ^21^, ^22^, ^23^, ^24^, ^25^, ^26^, ^27^, ^28^, ^29^, ^30^ ^]^ holiday junctions,^[^ ^36^, ^37^, ^38^, ^39^, ^40^, ^41^ ^]^ and double‐crossover‐like (DXL) motif,^[^ ^31^, ^42^ ^]^ and several DNA origami structures,^[^ ^43^, ^44^, ^45^, ^46^, ^47^ ^]^ have been successfully developed to assemble into designed 3D crystals. Some of those crystals can accommodate small molecules and allow structural study of the small molecule‐DNA interactions.^[^ ^31^, ^32^, ^33^, ^34^, ^35^ ^]^ However, the original goal of using engineered DNA crystals as hosts to arrange large biomacromolecules for structural study remains elusive. To achieve this goal, i) the DNA crystals have to have large empty voids to accommodate large guest molecules; ii) the guest molecules have to be incorporated into the crystals at defined both locations and orientations relative to the crystals; iii) the hybrid DNA‐guest crystals have to be ordered enough to diffract. Here, we report one effort in this direction to study the structure of DNA T‐junction,^[^ ^48^ ^]^ a commonly used DNA nanomotif.

A well‐studied, DNA tensegrity triangle crystal^[^ ^10^ ^]^ were explored as a crystal scaffold to regularly arrange DNA T‐junction motifs in 3D space (Figure 1). T‐junction is a T‐shaped, structural motif designed by Satoshi Murata and Hamada Shogo (Figure 1a).^[^ ^48^ ^]^ It is formed by hybridization between a single‐stranded DNA (ssDNA) sticky end (black) of a DNA duplex and a ssDNA bulge loop of another DNA duplex (green). The motif contains three component helical domains, named arms A, B, and C as defined by boxes with three different colors. The T‐junction motif provides both a programmable inter‐DNA tile cohesion and a three‐armed structural motif for DNA nanoconstruction.^[^ ^48^, ^49^, ^50^, ^51^, ^52^ ^]^ However, its structural detail, such as geometry and the rotational phases of each arm, has not been studied, though such information is critically needed for the fine control of the self‐assembly of complicated DNA involving T‐junctions.

**Figure 1:**
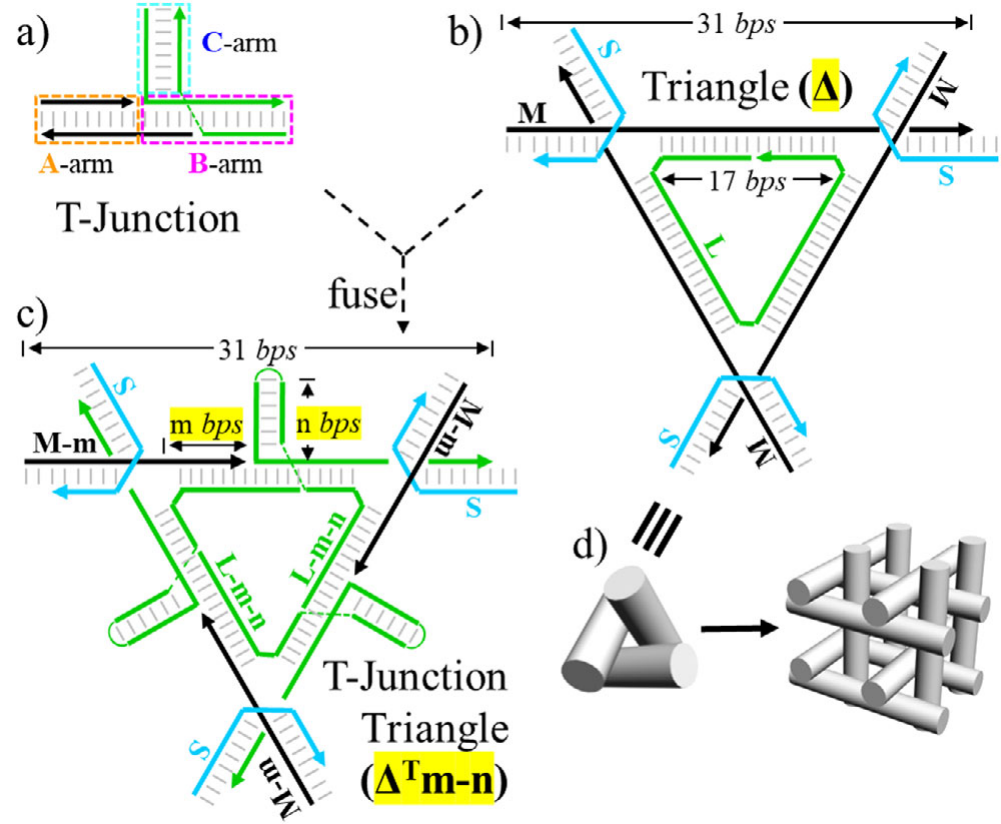
Arranging DNA T‐junctions into 3D crystals. a) scheme of a DNA T‐junction motif. b) scheme of a 3‐turn, symmetric, DNA tensegrity triangle (**Δ)**. c) scheme of a 3‐turn, symmetric, T‐junction tensegrity triangle (**Δ^T^m‐n)**. Note the definition of values of *m* and *n*. d) Assembly of tensegrity triangles into crystals via sticky‐end cohesion. Each rod represents a DNA duplex.

A symmetric, 3‐turn, DNA tensegrity triangle (named as **Δ**) consists of three inter‐connected, 3‐turn‐long, DNA duplexes (Figure 1b).^[^ ^10^ ^]^ Equipped with complementary sticky ends, the tensegrity triangles can associate with one another and propagate in three orthogonal directions to form 3D crystals (Figure 1d). For structural characterization, a T‐junction is incorporated at the center helical domain of each component duplex of the triangle, resulting in a modified, T‐junction triangle (**Δ^T^)** motif (Figure 1c). Both arms A and B are incorporated into the triangle duplexes, but arm C points out. The **Δ^T^** motif contains three T‐junctions and is composed of three long green strands (L), three short cyan strands (S), and three black medium strands (M). Strands in the same color are identical to each other and related to each other by a three‐fold rotational axis. The T‐junction triangle is named as **Δ^T^m‐n**. **Δ^T^** stands for T‐junction‐containing triangle; numbers m and n indicate the distance (in base pairs, bps) between the hairpin and the left corner of the triangle and the length (in bps) of the side duplex arm (C‐arm), respectively (Figure 1c).

We started the exploration with **Δ^T^7‐6** (Figure 2). The side C‐arm is a 6‐bp‐long hairpin and is 7 bps away from the left corner of the triangle (Figure 2a). The crystals were assembled according to literature.^[^ ^10^ ^]^ Briefly, equal molar ratio of the component DNA strands (Figure S1) were mixed in Mg^2+^‐containing buffer and annealed from 95 °C to 25 °C over 8 h. Then the annealed DNA samples was used for crystallization with hanging‐drop setup at 25 °C. Rhombohedral shape crystals with a size of ∼150 µm x 150 µm x 100 µm were observed after 3–5 days (Figure 2b). They had clear facets and sharp edges. Native polyacrylamide gel electrophoresis (nPAGE) confirmed that the DNA component strands, upon thermal annealing, associated with each other to form a triangle motif, a 3:3:3 complex (Figure 2c). To confirm that the crystals were indeed assembled from the triangle motifs, we dissolved the crystals under native condition and analyzed the DNA sample by nPAGE. A dominant, sharp band of triangle appeared with light fast‐moving smear, indicating that there was slight dissociation of the triangles during nPAGE that had lower ionic strength than the crystallization drop did. Actually, the triangle band from dissolved crystals was more dominant than that from the direct assembled DNA sample; indicating that the T‐junction crystals got greatly enriched by crystallization. It was not a surprise as crystallization was an efficient way for purification. Only molecules fit the crystal lattices could be incorporated into the crystals and other related species could not.

**Figure 2:**
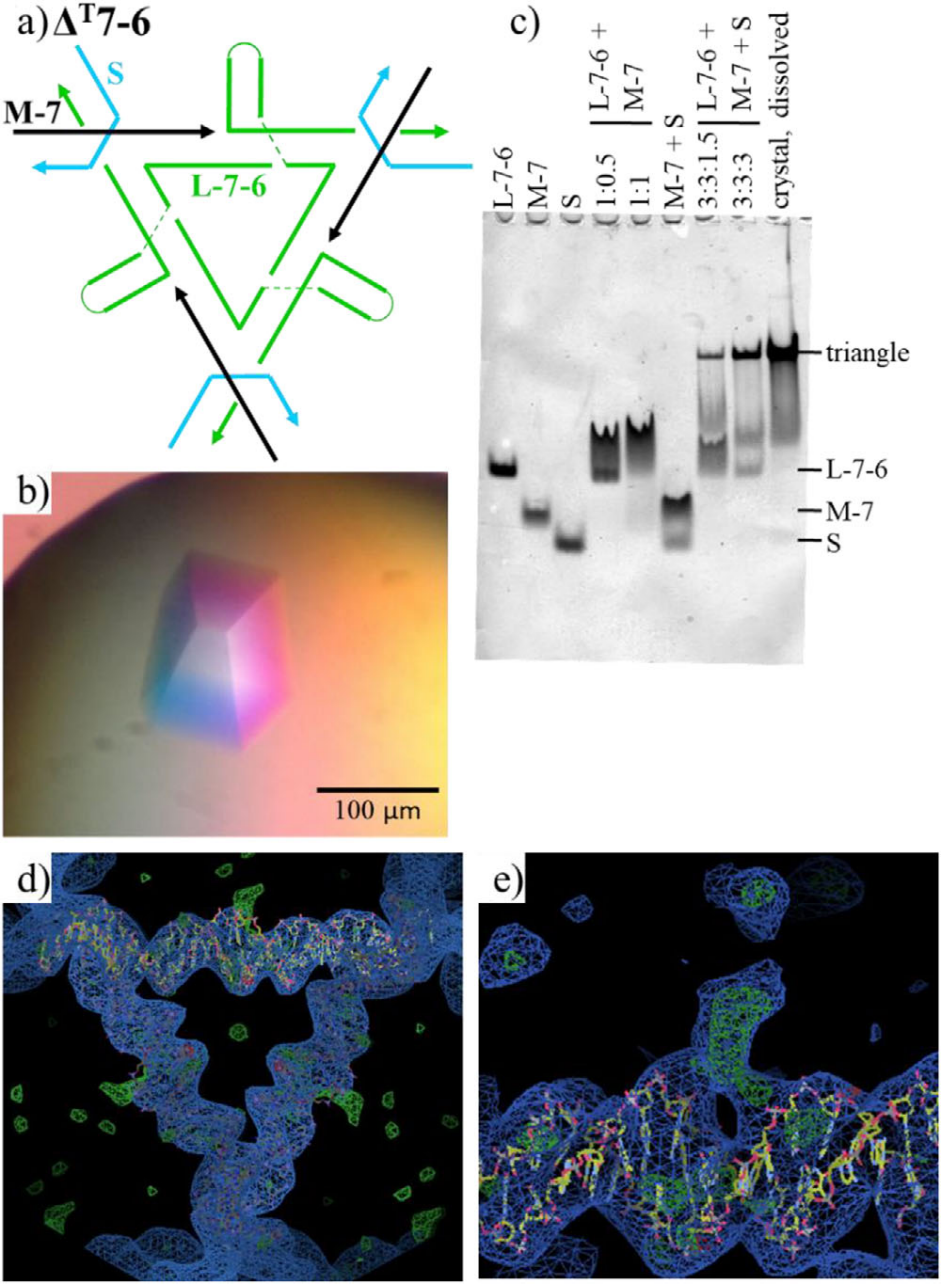
Crystallographic study of **Δ^T^7‐6**. a) scheme of a 3‐turn, T‐junction triangle **Δ^T^7‐6**. b) an optical image of the assembled DNA crystal. c) native polyacrylamide gel (6%) electrophoresis (nPAGE) analysis of the assembly of **Δ^T^7‐6** motif and crystals. The sample compositions and the chemical identify of each band are indicated above and the right side of the gel, respectively. d) Superimposing the electron density and a structure model (duplex). e) a closed‐up view of the T‐junction (electron density map and the duplex structural model). Note the extra electron density (green) corresponding to C‐arm.

The 3‐turn **Δ^T^7‐6** crystal was characterized by X‐ray crystallography. The crystal had a R3 rhombohedral lattice with a unit cell: *a* = *b* = *c* = 100.2 Å and α = β = γ = 113.2° (Table S1). The data matches with the parameters of conventional 3‐turn **Δ** crystal within 2% error in cell edge and angles, indicating overall the triangle structure remained the same after T‐junction was introduced.^[^ ^10^ ^]^ The structure of **Δ^T^7‐6** crystal was solved by molecular replacement (MR) method. A 31 bp duplex was applied as the initial search model for MR and then refined in PHENIX. The refinement result was shown in Table S2 and the output model for **Δ^T^7‐6** triangle was shown in Figure 2. Similar to conventional tensegrity triangle crystal lattices, any T‐junction triangle associated with six neighbors to form periodic 3D DNA framework. Different from the original design, For the **Δ^T^7‐6**, at the duplex middle where the T‐junction locates, extra densities (in green color) corresponding to the C‐arm were observed (Figure 2d,e). The clear, extra electron density indicated that the T‐junction was relatively rigid, well‐defined structure. Otherwise, the electron density would smear out and could not be detected. Model building was then carried out for the missing hairpin. Due to the flexibility of the hairpin loop, only the 6‐bp‐long, duplex stem of the C‐arm was built. The built model was further refined to generate output model for **Δ^T^7‐6**. From the refined result (Figure S2), the T‐junction structure was close to a T‐shape and the A‐arm and B‐arm in T‐junction were coaxially arranged and the C‐arm pointed out from the A‐B arm duplex. The crystallographically observed T‐junction show slightly bending at the 3‐way junction point; thus, not a right‐angled T shape. This result was consistent with the previous AFM characterization of T‐junction motif in assembled 2D arrays.^[^ ^48^, ^49^, ^50^, ^51^, ^52^ ^]^

The 3‐turn tensegrity triangle crystal is quite porous and has large internal cavities. Such cavities can accommodate T‐junctions of different‐sized side C‐arms at different locations of the triangle (Figure 3). To demonstrated such versatility, we prepared two series of T‐junction triangles (**Δ^T^**): i) Set the hairpin length *n* = 6 bps and varying *m* to be 6, 7, 8 bps (**Δ^T^6‐6**, **Δ^T^7‐6**, and **Δ^T^8‐6**); and ii) elongating the side hairpin length *n* to 11 bps and varying *m* to be 6, 7, 8 bps (**Δ^T^6‐11**, **Δ^T^ 7–11**, and **Δ^T^8‐11**) (Figures S2–S7). Systematic experimental characterization of these two series of DNA **Δ^T^** crystals would allow us to illustrate the impact of different factors on the T‐junction triangle (**Δ^T^**) and validate the T‐junction structure.

**Figure 3:**
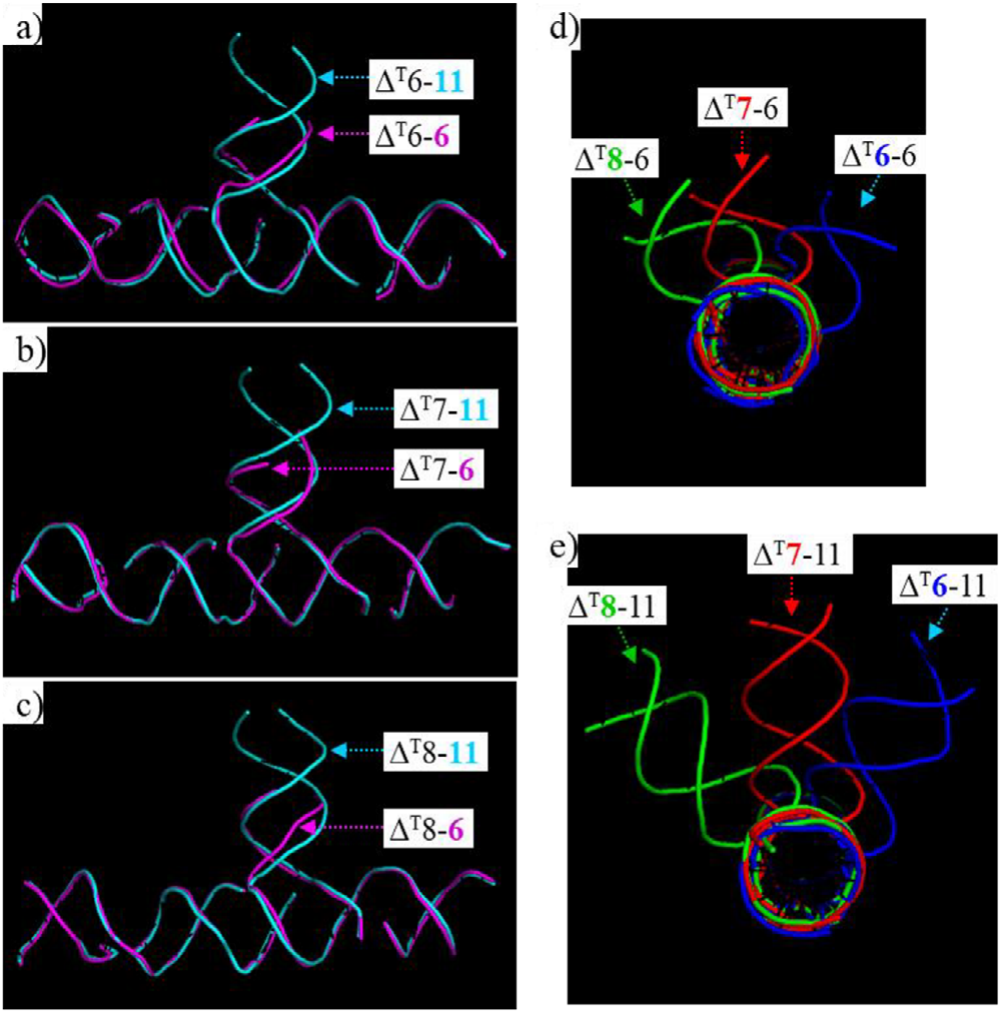
Summary of the structures of T‐junction triangle (**Δ^T^**) crystals with different length T‐junctions at different locations. a)–c) Superimposing the **Δ^T^** variants with C‐arms at the same position but at different arm lengths. d) and e) Superimposing the **Δ^T^** variants with C‐arms at different locations but the same arm length.

Experimental protocol for characterizing these two series of designs is the same as that for characterizing **Δ^T^7‐6** triangle. Crystals for all designs showed up under the same crystallization condition as **Δ^T^7‐6** crystal with similar sizes (∼150 µm x 150 µm x 100 µm for the largest crystals, Figures S2–S7), suggesting that the overall crystal lattices remained identical. In addition, it demonstrated one advantage of engineered DNA crystals: they can significantly reduce the effort on testing crystallization conditions and minimizing the required sample amounts.

nPAGEs (Figures S2–S7) confirmed that all these T‐junction triangles could readily assembled upon thermal annealing. The primary DNA complexes from dissolved crystals were indeed the T‐junction crystals. As observed for **Δ^T^7‐6**, these triangle variants also show stronger bands from the dissolved crystals than those from directly assembled samples.

The crystals of all the **Δ^T^** variants were studied by X‐ray crystallography (Table S1 and Figures S2–S7). They all shared R3 rhombohedral lattices with the same unit cell dimensions: *a* = *b* = *c* ∼100 Å and α = β = γ = 112 – 114°. The data matched with the parameters of conventional 3‐turn tensegrity triangle crystals within 3% error; indicating that the overall DNA triangle frameworks remained the same for all **Δ^T^** variants. The resolutions of the crystals ranged from 6.1 to 7.2 Å. The crystal structures were solved by MR method with a 31 bp B‐DNA duplex as the search model and further refined in PHENIX. Since a 31 bp duplex is only one edge of the triangle and the C‐arms in the T‐junctions are missing in the model, we expect to observe extra densities for C‐arms in the Fo‐Fc map. Indeed, for all **Δ^T^** variants, extra densities (colored green) could clearly be identified (Figure 2d,e for **Δ^T^7‐6**, S3d and S3e for **Δ^T^6‐6**, S4d and S4e for **Δ^T^6‐11**, S5d and S5e for **Δ^T^7‐11**, S6d and S6e for **Δ^T^8‐6**, S7d and S7e for **Δ^T^8‐11**). In **Δ^T^6‐6** (Figure S3d,e) and **Δ^T^6‐11** (Figure S4d,e), the extra densities were directly below the triangle planes. To clearly show those extra densities, all the rendered images were flipped 180° horizontally from the schemes of the secondary structures (Figure S1) for all later on model display. So, the original right shift of C‐arm positions will be shown as left shift in the models and vice versa. Matched well with the predictions from the designs, the positions of the extra densities in Fo‐Fc map changed with the designs. For crystals with the same C‐arm positions, the orientations and the locations of the extra C‐arms were almost identical (Figure 3a–c). The above result suggested that the observed extra densities indeed corresponded to the C‐arms in T‐junctions. Model buildings were further carried out, similarly as described in **Δ^T^7‐6** data process, by fitting Coot‐generated, idealized B‐DNA duplexes (C‐arms) into the electron density map. The built models were further refined in PHENIX and the results were shown in Figures S2–S7. The refinement statistics of all **Δ^T^** variants were listed in Table S2. Crystals of all **Δ^T^** variants have essentially the same frameworks (Figures 4, S8, and S9), which were inherited from the original, 3‐turn, tensegrity triangle crystal.

**Figure 4:**
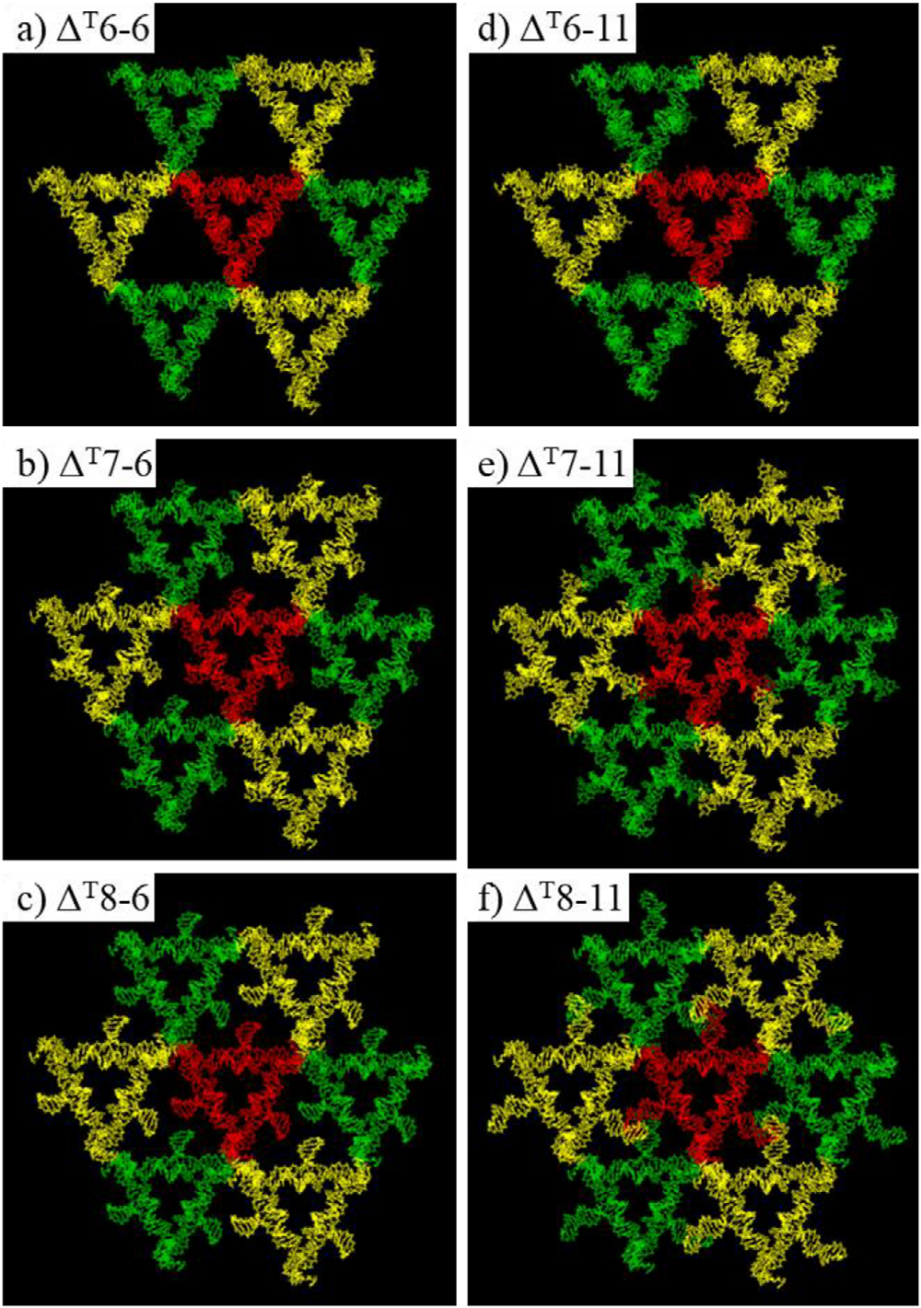
Front views of the arrangement of Δ^T^ in crystals. a) Δ^T^6‐6, b) Δ^T^7‐6, c) Δ^T^8‐6, d) Δ^T^6‐11, e) Δ^T^7‐11, and f) Δ^T^8‐11. The center red Δ^T^ connects with both yellow and green Δ^T^s through one edge. The yellow Δ^T^s locate at the plane close to the viewer and the green Δ^T^s locate at the plane further away from the viewer. All crystals exhibited same DNA framework structures which was inherited from the original tensegrity triangle crystal.

We compared the structures of all the **Δ^T^** variants with either the same C‐arm position or same length (Figure 3d,e). When the C‐arm position was the same (Figure 3a–c), the triangles could pretty much completely overlap with each other, **Δ^T^6‐6**/**Δ^T^6‐11** (Figure 3a), **Δ^T^7‐6**/**Δ^T^7‐11** (Figure 3b), and **Δ^T^8‐6**/**Δ^T^8‐11** (Figure 3c), except the C‐arm lengths. More interesting was superimposing the triangles with C‐arms of the same length but different C‐arm positions, **Δ^T^6‐6**/**Δ^T^7‐6**/**Δ^T^8‐6**/ (Figure 3d) and **Δ^T^6‐11**/**Δ^T^7‐11**/**Δ^T^8‐11**/ (Figure 3e). By looking along the triangle edge duplex, the C‐arms rotated stepwise by 28°–54° (rotating angles from **Δ^T^6‐6** to **Δ^T^7‐6** to **Δ^T^8‐6** are 28° and 54°, respectively; rotating angles from **Δ^T^6‐11** to **Δ^T^7‐11** to **Δ^T^8‐11** are 43° and 37°, respectively), consistent with the twist of 34°/bp for B‐DNA duplex. This study show that the T‐junction was relatively rigid and had a conservative structure. The position and orientation of T‐linkage could be predicted from the design. Thus, the T‐junction‐containing crystals could potentially be applied to arrange guest molecules into DNA 3D frameworks with precise organization of both position and orientation by controlling the position and length of the C‐arm.

In conclusion, we have successfully crystallographically studied T‐junction structure by arranging it into engineered DNA crystals. The solved structures are consistent with all previous studies.^[^ ^48^, ^49^, ^50^, ^51^, ^52^ ^]^ We would like to point out the crystals based on DNA triangles are very robust (Figure. S10). DNA molecules are not limited to any special DNA sequences (except the four base pairs flanking each junction point). The crystals grow in common biological buffers (pH 5.5–8.5) and are compatible with different cations Na^+^, K^+^, Li^+^, Ca^2+^, Mg^2+^, and ammonium. The significance of this study is two‐folded. i) It experimentally demonstrated that engineered DNA crystals indeed could provide a platform to organize biomolecules into regular arrangement for structural study, as proposed by Dr. Nadrian Seeman 40 years ago.^1^ ii) The C‐arm in T‐junction is well ordered in the crystals, but not part of the crystal frameworks. Thus, the C‐arm provides an excellent anchor for introduction of guest molecules with precise position and orientation control relative to the crystal lattice.^[^ ^53^ ^]^

## Supporting Information

Materials and detailed experimental methods; figures for additional characterization of DNA crystals; and summary of DNA crystallography.

## Conflict of Interests

The authors declare no conflict of interest.

## Supporting information



Supporting Information

Supporting Information

## Data Availability

Molecular structures were deposited to the Protein Databank (https://www.rcsb.org/) under accession codes: 9DFY (**Δ^T^6‐6**), 9DFZ (**Δ^T^6‐11**), 9DG0 (**Δ^T^7‐6**), 9DGN (**Δ^T^7‐11**), 9DGL (**Δ^T^8‐6**), and 9DGM (**Δ^T^8‐11**).

## References

[1] N. C. Seeman , J. Theor. Biol. 1982, 99, 237–247, 10.1016/0022-5193(82)90002-9. 6188926

[2] N. C. Seeman , Nature 2003, 421, 427–431, 10.1038/nature01406. 12540916

[3] A. V. Pinheiro , D. Han , W. M. Shih , Y. Hao , Nat. Nanotech. 2011, 6, 763–772, 10.1038/nnano.2011.187. PMC3334823 22056726

[4] M. R. Jones , N. C. Seeman , C. A. Mirkin , Science 2015, 347, 1260901, 10.1126/science.1260901. 25700524

[5] N. C. Seeman , H. F. Sleiman , Nat. Rev. Mater. 2018, 3, 17068, 10.1038/natrevmats.2017.68.

[6] P. W. K. Rothemund , Nature 2006, 440, 297–302, 10.1038/nature04586. 16541064

[7] B. Wei , M. Dai , P. Yin , Nature 2012, 485, 623–626, 10.1038/nature11075. 22660323 PMC4238960

[8] G. Tikhomirov , P. Petersen , L. Qian , Nature 2017, 552, 67–71, 10.1038/nature24655. 29219965

[9] S. Jiang , F. Zhang , H. Yan , Nat. Mater. 2020, 19, 694–700, 10.1038/s41563-020-0719-3. 32581353

[10] J. Zheng , J. J. Birktoft , Y.i Chen , T. Wang , R. Sha , P. E. Constantinou , S. L. Ginell , C. Mao , N. C. Seeman , Nature 2009, 461, 74–77, 10.1038/nature08274. 19727196 PMC2764300

[11] Y. Zhao , A. R. Chandrasekaran , D. A. Rusling , K. Woloszyn , Y. Hao , C. Hernandez , S. Vecchioni , Y. P. Ohayon , C. Mao , N. C. Seeman , R. Sha , J. Am. Chem. Soc. 2023, 145, 3629–3636 . 10.1021/jacs.2c12667 PMC10032566 36731121

[12] B. Lu , K. Woloszyn , Y. P. Ohayon , B. Yang , C. Zhang , C. Mao , N. C. Seeman , S. Vecchioni , R. Sha , Angew. Chem. Int. Ed. 2023, 62, e202213451, 10.1002/anie.202213451. 36520622

[13] S. Vecchioni , B. Lu , W. Livernois , Y. P. Ohayon , J. B. Yoder , C.‐F. Yang , K. Woloszyn , W. Bernfeld , M. P. Anantram , J. W. Canary , W. A. Hendrickson , L. J. Rothschild , C. Mao , S. J. Wind , N. C. Seeman , R. Sha , Adv. Mater. 2023, 35, e2210938, 10.1002/adma.202210938. 37268326

[14] B. Lu , S. Vecchioni , Y. P. Ohayon , K. Woloszyn , T. Markus , C. Mao , N. C. Seeman , J. W. Canary , R. Sha , Small 2022, 18, 2205830 . 10.1002/smll.202205830 36408817

[15] B. Lu , Y. P. Ohayon , K. Woloszyn , C.‐F. Yang , J. B. Yoder , L. J. Rothschild , S. J. Wind , W. A. Hendrickson , C. Mao , N. C. Seeman , J. W. Canary , R. Sha , S. Vecchioni , J. Am. Chem. Soc. 2023, 145, 17945–17953, 10.1021/jacs.3c05478. 37530628

[16] S. Vecchioni , Y. P. Ohayon , C. Hernandez , S. Hoshika , C. Mao , S. A. Benner , R. Sha , Nano Lett. 2024, 24, 14302–14306, 10.1021/acs.nanolett.4c03949. 39471314 PMC11566107

[17] K. Woloszyn , S. Vecchioni , Y. P. Ohayon , B. Lu , Y. Ma , Q. Huang , E. Zhu , D. Chernovolenko , T. Markus , N. Jonoska , C. Mao , N. C. Seeman , R. Sha , Adv. Mater. 2022, 34, 2206876, 10.1002/adma.202206876. 36100349

[18] J. Lyu , T. Zhu , Y. Zhou , T. Zhao , M. Fei , X. Zhong , H. He , Chemistry 2024, 30, e202400012, 10.1002/chem.202400012. 38477176

[19] J. Chen , Z. Dai , H. Lv , Z. Jin , Y. Tang , X. Xie , J. Shi , F. Wang , Q. Li , X. Liu , C. Fan , Proc. Natl. Acad. Sci. USA 2024, 121, e2312596121, 10.1073/pnas.2312596121. 38437555 PMC10945798

[20] J. Chen , M. Li , Z. Li , Y. Tang , H. Zhang , J. Cheng , Z. Dai , X. Zuo , Q. Li , F. Wang , S. Jia , H. Lv , C. Fan , X. Liu , Angew. Chem. Int. Ed. 2025 64, e202511377. 10.1002/anie.202511377 40755298

[21] Y. Hao , M. Kristiansen , R. Sha , J. J. Birktoft , C. Hernandez , C. Mao , N. C. Seeman , Nat. Chem. 2017, 9, 824–827, 10.1038/nchem.2745. 28754940

[22] Y. P. Ohayon , C. Hernandez , A. R. Chandrasekaran , X. Wang , H. O. Abdallah , M. A. Jong , M. G. Mohsen , R. Sha , J. J. Birktoft , P. S. Lukeman , P. M. Chaikin , S. L. Ginell , C. Mao , N. C. Seeman , ACS Nano 2019, 13, 7957–7965, 10.1021/acsnano.9b02430. 31264845 PMC6660133

[23] B. Lu , S. Vecchioni , Y. P. Ohayon , R. Sha , K. Woloszyn , B. Yang , C. Mao , N. C. Seeman , ACS Nano 2021, 15, 16788–16793, 10.1021/acsnano.1c06963. 34609128

[24] D. A. Rusling , A. R. Chandrasekaran , Y. P. Ohayon , T. Brown , K. R. Fox , R. Sha , C. Mao , N. C. Seeman , Angew. Chem. Int. Ed. 2013, 52, 10553–10556 . 10.1002/anie.201309914 PMC4037404 24615910

[25] T. Wang , R. Sha , J. Birktoft , J. Zheng , C. Mao , N. C. Seeman , J. Am. Chem. Soc. 2010, 132, 15471–15473, 10.1021/ja104833t. 20958065 PMC2975597

[26] M. Zheng , Z. Li , C. Zhang , N. C. Seeman , C. Mao , Adv. Mater. 2022, 34, 2200441, 10.1002/adma.202200441. 35389546

[27] Z. Li , M. Zheng , L. Liu , N. C. Seeman , C. Mao , J. Am. Chem. Soc. 2021, 143, 14987–14991, 10.1021/jacs.1c07279. 34516099

[28] J. Zhao , Y. Zhao , Z. Li , Y. Wang , R. Sha , N. C. Seeman , C. Mao , Angew. Chem. Int. Ed. 2018, 57, 16529–16532, 10.1002/anie.201809757. 30240115

[29] J. Zhao , A. R. Chandrasekaran , Q. Li , X. Li , R. Sha , N. C. Seeman , C. Mao , Angew. Chem. Int. Ed. 2015, 54, 9936–9939, 10.1002/anie.201503610. 26136359

[30] Z. Li , L. Liu , M. Zheng , J. Zhao , N. C. Seeman , C. Mao , J. Am. Chem. Soc. 2019, 141, 15850–15855, 10.1021/jacs.9b06613. 31553173

[31] C. Zhang , J. Zhao , B. Lu , N. C. Seeman , R. Sha , N. Noinaj , C. Mao , J. Am. Chem. Soc. 2023, 145, 4441–4448 . 10.1021/jacs.3c00081 36791277

[32] C. R. Simmons , A. Buchberger , S. J. W. Henry , A. Novacek , N. E. Fahmi , T. MacCulloch , N. Stephanopoulos , H. Yan , J. Am. Chem. Soc. 2023, 145, 26075–26085, 10.1021/jacs.3c07802. 37987645 PMC10789492

[33] P. H. Winegar , O. G. Hayes , J. R. McMillan , C. Adrian Figg , P. J. Focia , C. A. Mirkin , Chem 2020, 6, 1007–1017, 10.1016/j.chempr.2020.03.002. 33709040 PMC7946157

[34] A. R. Orun , E. T. Shields , S. Dmytriw , A. Vajapayayula , C. K. Slaughter , C. D. Snow , ACS Nano 2023, 17, 13110–13120, 10.1021/acsnano.2c07282. 37407546 PMC10373652

[35] Y.e Tian , J. R. Lhermitte , L. Bai , T. Vo , H. L. Xin , H. Li , R. Li , M. Fukuto , K. G. Yager , J. S. Kahn , Y. Xiong , B. Minevich , S. K. Kumar , O. Gang , Nat. Mater. 2020, 19, 789–796, 10.1038/s41563-019-0550-x. 31932669

[36] C. R. Simmons , F. Zhang , J. J. Birktoft , X. Qi , D. Han , Y. Liu , R. Sha , H. O. Abdallah , C. Hernandez , Y. P. Ohayon , N. C. Seeman , H. Yan , J. Am. Chem. Soc. 2016, 138, 10047–10054, 10.1021/jacs.6b06508. 27447429

[37] C. R. Simmons , F. Zhang , T. MacCulloch , N. Fahmi , N. Stephanopoulos , Y. Liu , N. C. Seeman , Y. Hao , J. Am. Chem. Soc. 2017, 139 11254–11260, 10.1021/jacs.7b06485. 28731332

[38] F. Zhang , C. R. Simmons , J. Gates , Y. Liu , H. Yan , Angew. Chem. Int. Ed. 2018, 57, 12504–12507, 10.1002/anie.201807223. 30066355

[39] C. R. Simmons , T. MacCulloch , F. Zhang , Y. Liu , N. Stephanopoulos , Y. Hao , Angew. Chem. Int. Ed. 2020, 59, 18619–18626, 10.1002/anie.202005505. 32533629

[40] C. R. Simmons , T. MacCulloch , M. Krepl , M. Matthies , A. Buchberger , I. Crawford , J. Šponer , P. Šulc , N. Stephanopoulos , H. Yan , Nat. Commun. 2022, 13, 3112, 10.1038/s41467-022-30779-6. 35662248 PMC9166708

[41] J. Zhao , C. Zhang , B. Lu , R. Sha , N. Noinaj , C. Mao , J. Am. Chem. Soc. 2023, 145, 10475–10479, 10.1021/jacs.3c01941. 37134185

[42] C. Zhang , V. E. Paluzzi , R. Sha , N. Jonoska , C. Mao , Adv. Mater. 2023, 35, e2302345, 10.1002/adma.202302345. 37220213

[43] T. Zhang , C. Hartl , K. Frank , A. Heuer‐Jungemann , S. Fischer , P. C. Nickels , Adv. Mater. 2018, 30, 1800273 . 10.1002/adma.201800273 29774971

[44] G. Posnjak , X. Yin , P. Butler , O. Bienek , M. Dass , S. Lee , I. D. Sharp , T. Liedl , Science 2024, 384, 781–785 . 38753795 10.1126/science.adl2733 PMC7616107

[45] H. Liu , M. Matthies , J. Russo , L. Rovigatti , R. Pradeep Narayanan , T. Diep , D. McKeen , O. Gang , N. Stephanopoulos , F. Sciortino , H. Yan , F. Romano , P. Šulc , Science 2024, 384, 776–781, 10.1126/science.adl5549. 38753798

[46] L. Dai , X. Hu , M. Ji , Y.e Tian , Proc. Natl. Acad. Sci. USA 2023, 120, e2302142120, 10.1073/pnas.2302142120. 37399399 PMC10334761

[47] F. Hong , S. Jiang , T. Wang , Y. Liu , H. Yan , Angew. Chem. Int. Ed. 2016, 55, 12832–12835, 10.1002/anie.201607050. 27628457

[48] S. Hamada , S. Murata , Angew. Chem. Int. Ed. 2009, 48, 6820–6823, 10.1002/anie.200902662. 19688799

[49] Q.i Yang , X.u Chang , J. Y. Lee , M. Saji , F. Zhang , Nat. Commun. 2023, 14, 7675, 10.1038/s41467-023-43558-8. 37996416 PMC10667507

[50] T. Zhang , B. Wei , J. Am. Chem. Soc. 2023, 143, 16693–16699 2021. 10.1021/jacs.1c07824. 34606714

[51] X. Li , C. Zhang , C. Hao , C. Tian , G. Wang , C. Mao , ACS Nano 2012, 6, 5138–5142, 10.1021/nn300813w. 22559206

[52] J. Shin , J. Kim , S. H.a Park , T. H. Ha , ACS Nano 2018, 12, 9423–9432, 10.1021/acsnano.8b04639. 30114364

[53] X. Li , C. Zhang , C. Hao , C. Tian , G. Wang , C. Mao , ACS Nano 2012, 6, 5138–5142 . 22559206 10.1021/nn300813w

