# Supplementary material for: Crystallographic Study of DNA T‐Junction via Crystal Engineering
Source: Angew Chem Int Ed Engl. 2025 Oct 30;64(51):e18174. doi: 10.1002/anie.202518174 (PMC12707366; doi:10.1002/anie.202518174)

**Supporting Information**

**Material and Methods**

**DNA oligonucleotides.** DNA sequences were designed by the SEQUIN computer program. All DNA strands were purchased from the IDT and purified by 15-20% denaturing PAGE followed by desalting. The sequence of all strands are listed below:

L: CGGTATTCACCACGATGCGGTATTCACCACGATGCGGTATTCACCACGATG;

L-6-6: GCGAGTATTCACCGGCT CCGCGG TTTT CCGCGG CTCGCAGCCGGACAGCG;

L-6-11: GCGAGTATTCACCGGCT CCGCGGACAACTTTTGTTGTCCGCGG CTCGCAGCCGGACAGCG;

L-7-6: TGCGAGTATTCACCGGC CCGCGG TTTT CCGCGG TCGCAGCCGGACAGCG;

L-7-11: TGCGAGTATTCACCGGC CCGCGGACAACTTTTGTTGTCCGCGG TCGCAGCCGGACAGCG

L-8-6: CTGCGAGTATTCACCGG CCGCGG TTTT CCGCGG CGCAGCCGGACAGCG;

L-8-11: CTGCGAGTATTCACCGG CCGCGGACAACTTTTGTTGTCCGCGG CGCAGCCGGACAGCG;

M: CAGCAGCCTGAATACCGCATCGTGGACAGCG

M-6: CAGCAGCCTGAATA;

M-7: CAGCAGCCTGAATAC;

M-8: CAGCAGCCTGAATACT;

S: TGCGCTGTGGCTGC;

(underlined sequences are the hairpins in T-junctions).

**DNA triangle motifs:**

Δ: L + M + S (molar ratio: 1:3:3)

Δ^T^6-6: L-6-6 + M-6 + S (molar ratio: 1:1:1)

Δ^T^6-11: L-6-11 + M-6 + S (molar ratio: 1:1:1)

Δ^T^7-6: L-7-6 + M-7 + S (molar ratio: 1:1:1)

Δ^T^7-11: L-7-11 + M-7 + S (molar ratio: 1:1:1)

Δ^T^8-6: L-8-6 + M-8 + S (molar ratio: 1:1:1)

Δ^T^8-11: L-8-11 + M-8 + S (molar ratio: 1:1:1)

**Formation of DNA complexes and crystallization.** For all crystal designs, DNA strands were mixed according to the indicated ratio from the scheme in a Tris-acetic-EDTA-Mg^2+^ (TAE/Mg^2+^) buffer (8 mM Tris base, 4 mM acetic acid, 0.4 mM EDTA and 2.5mM magnesium acetate). Upon mixing, the sample solutions were first heated to 95 °C for 5 min, then stayed at 65 °C, 50 °C, 37 °C and 22 °C for 2 hours at each temperature. Then sample solution was mixed with the growing buffer (0.001 M Magnesium chloride hexahydrate, 0.005 M Tris hydrochloride, pH=7.5, 0.16 M Ammonium sulfate, 0.23 M sucrose) at a ratio of 4:5 (v/v) and grew against 1.6 M (NH_4_)_2_SO_4_ growing buffer by hanging drop method at room temperature. After 2-5 days, the drops shrank by about 90% and rhombohedral shaped crystals were obtained with dimensions up to 150 × 150 × 100 μm. The reservoir buffer concentration was further increased to 2.8 M (NH_4_)_2_SO_4_ and incubated overnight before characterized by X-ray diffraction.

**Native PAGEs.** Native PAGEs containing 6% polyacrylamide (19:1 acrylamide/bisacrylamide) were run on a SE600 cooled vertical electrophoresis unit (Hoefer, 250 V, constant voltage) at 4°C in TAE/Mg^2+^ buffer. After electrophoresis, the gels were stained with Stain-all dye (Sigma), distained by light and scanned by an office HP scanner.

**X-ray data collection and processing.** For each crystal design, a dataset of 180 degree X-ray diffraction was collected at 1.54 Å on a home source X-ray machine (R-axis IV, Hockmeyer Hall, Purdue) with exposure time of 10 min per frame per 1.5 degree oscillation angle. The collected diffraction data were processed with HKL-2000. After autoindexing, R3 space group is chosen according to the design for the integration, scaling and post-refinement before merging all data into the output sca file.

**Phase determination, model building and refinement.** Molecular replacement and structural refinement were carried out in PHENIX. Each diffraction dataset was first analyzed by Xtriage to evaluate data quality. Then an idealized, 31-bp-long, B-DNA duplex was used as a search model to perform molecular replacement and the result was first carried out by rigid body refinement followed by TLS refinement. Then xyz coordinated and group B-factor refinements were used. Basepairing restraint and auto optimized weight given by the software were applied during the refinement process. Extra densities in Fo-Fc map were observed (green densities) at the expected hairpin part for T-junction. A duplex pdb file with desired length was built and fit into the extra densities for each design and merged with the 31 bp duplex to build the new model. With the newly built models, the same refinement as described above was carried out to get final output models.

**Structural comparisons between different T-junction crystal designs and model display.** The models with same C-arm position and length were overlapped by least linear fitting with corresponding triangle edges. All the solved structures were displayed in COOT and PYMOL.


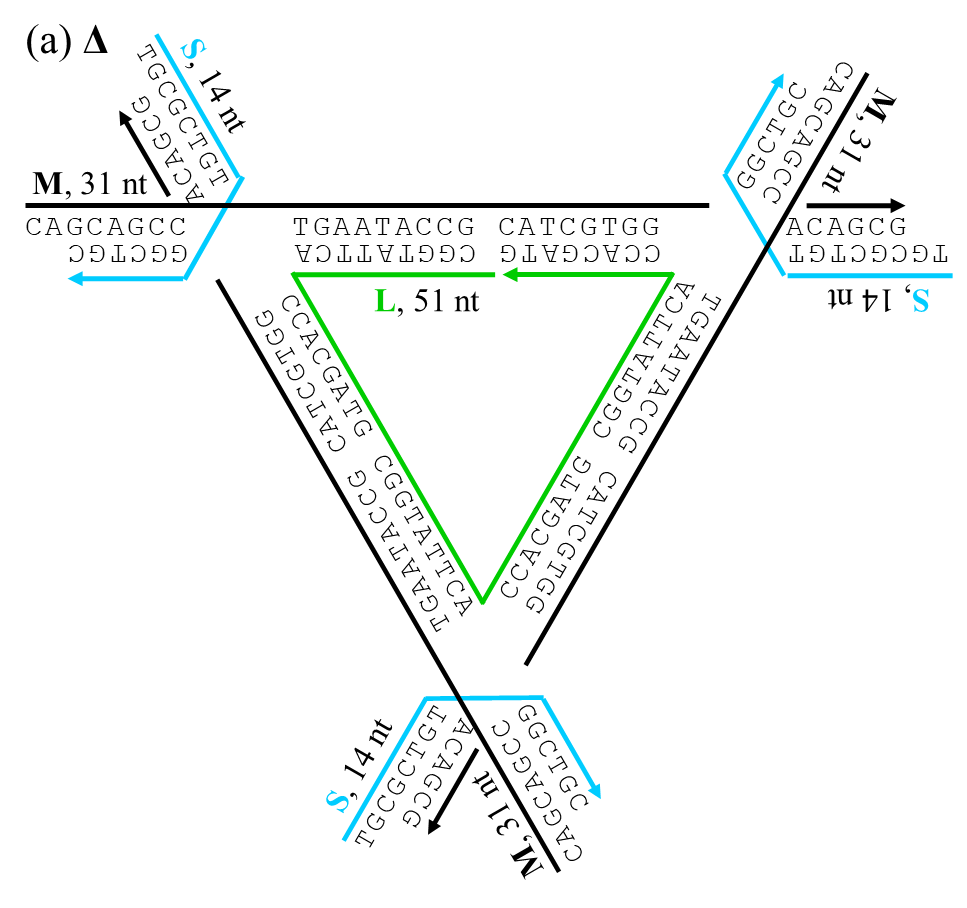


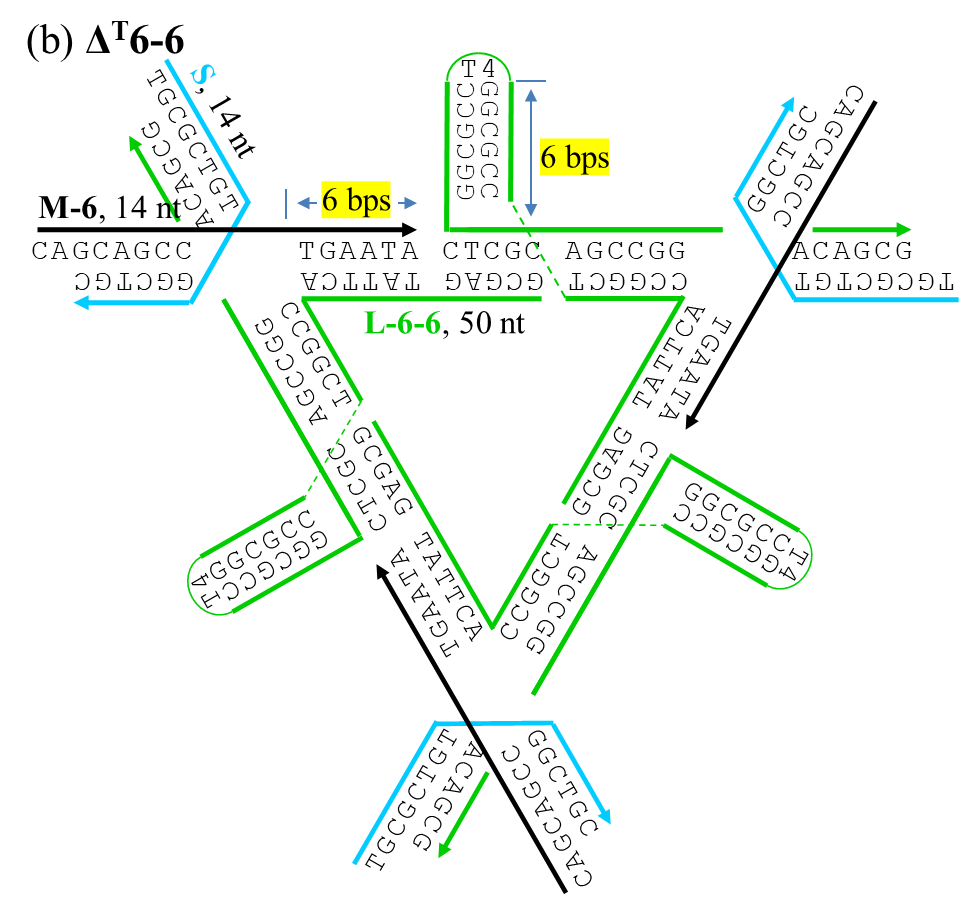

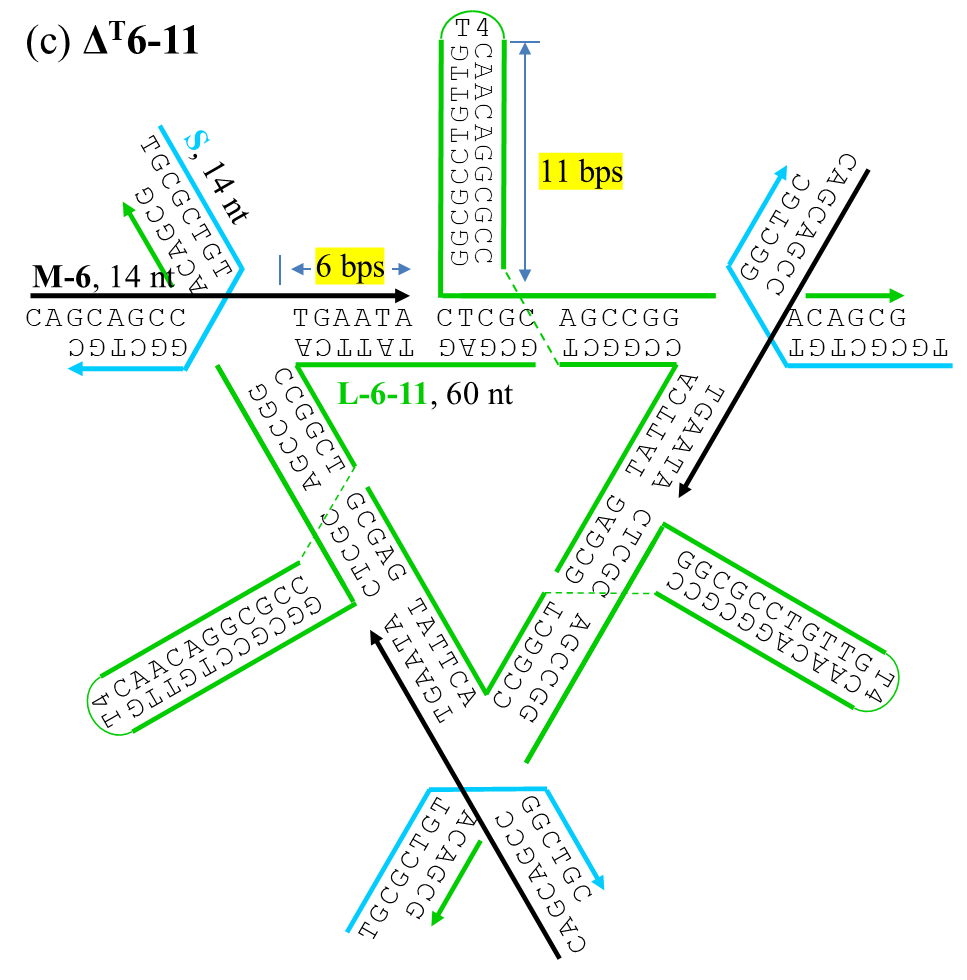


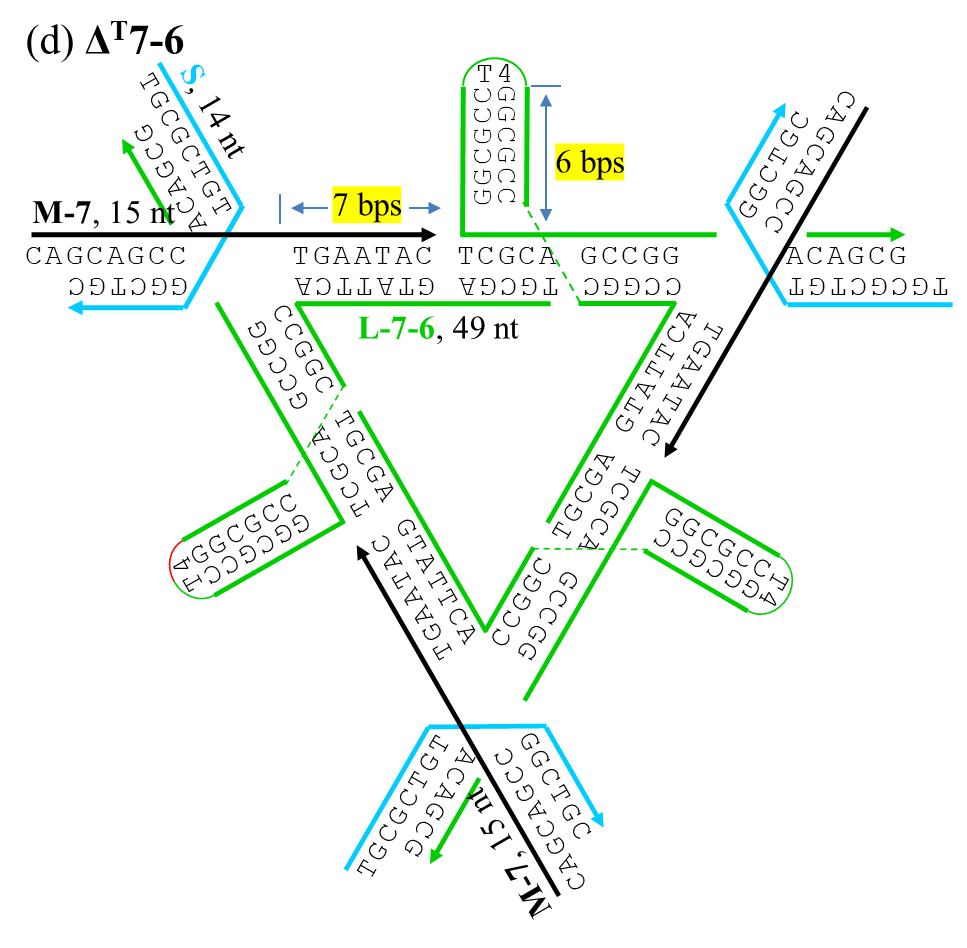

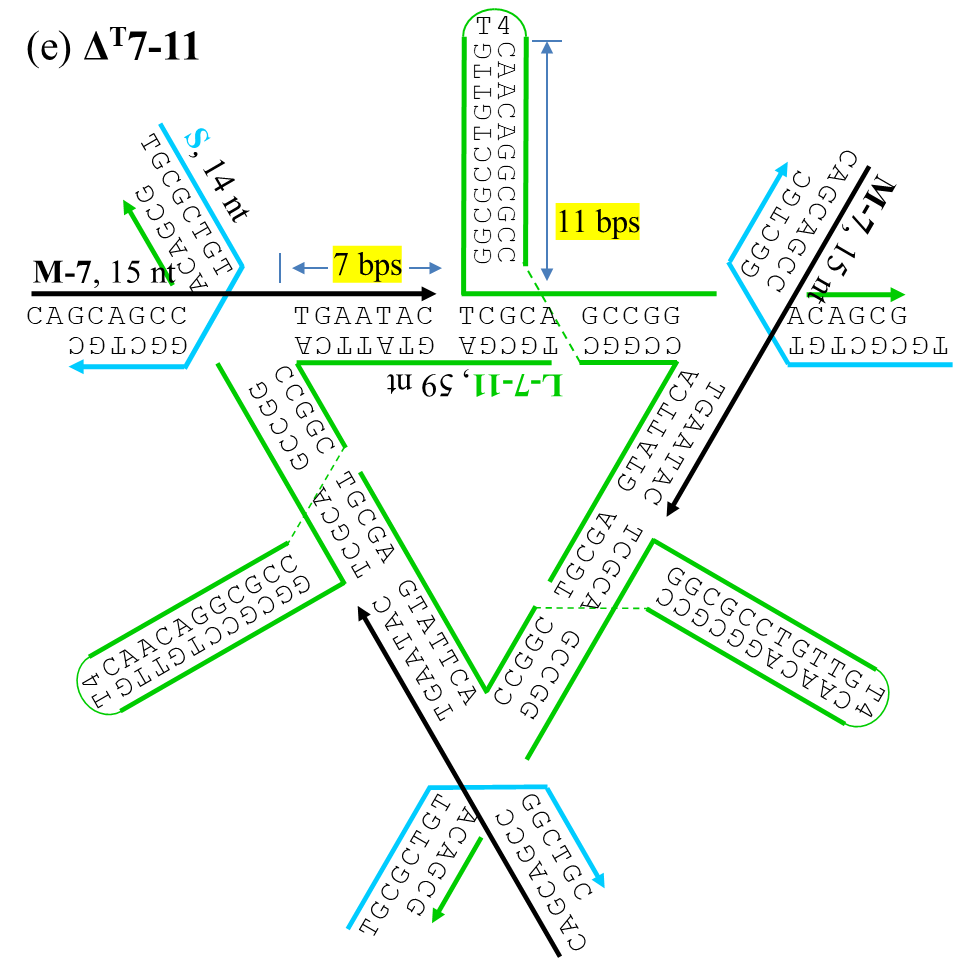


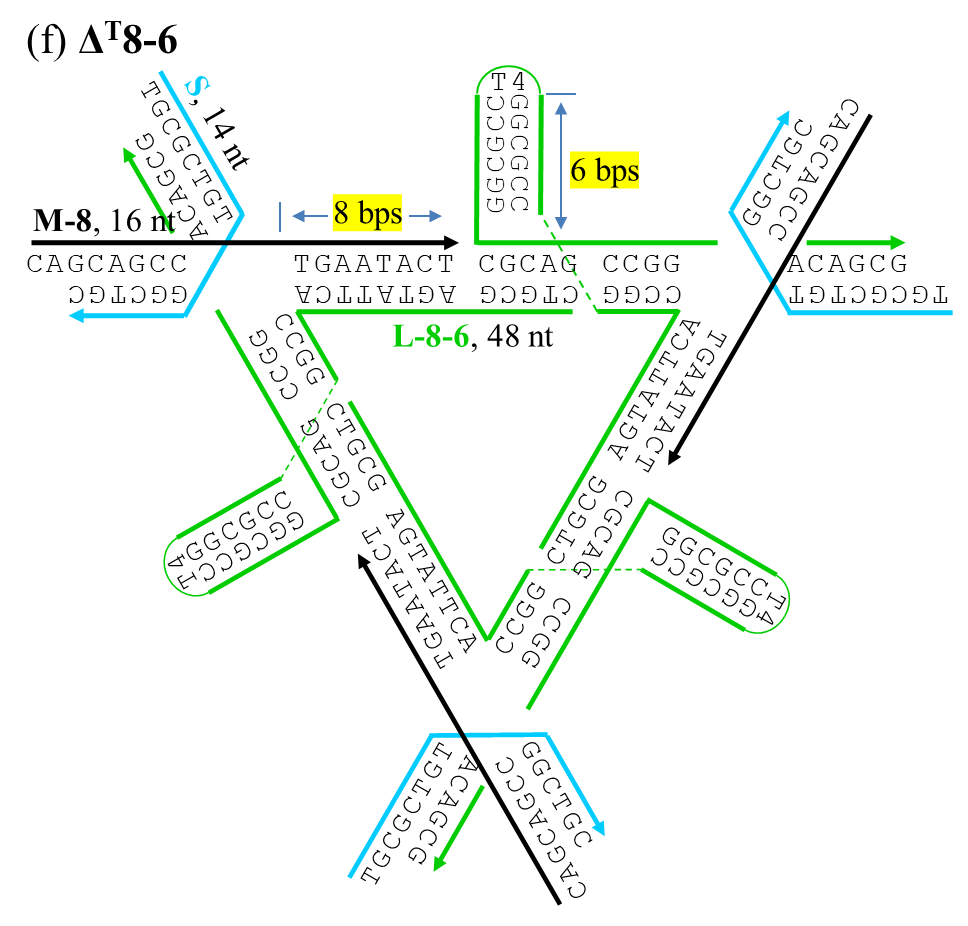

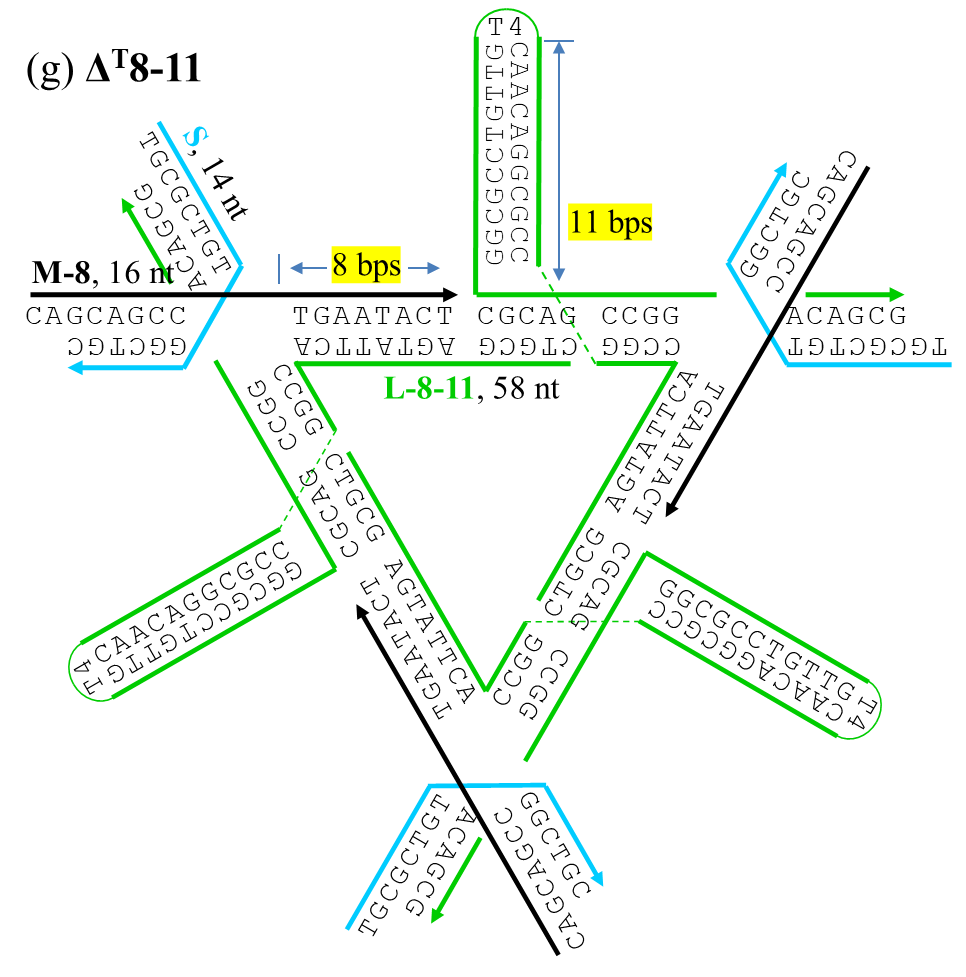


**Figure S1.** Schemes of the secondary structures of all triangle motifs used in this study.


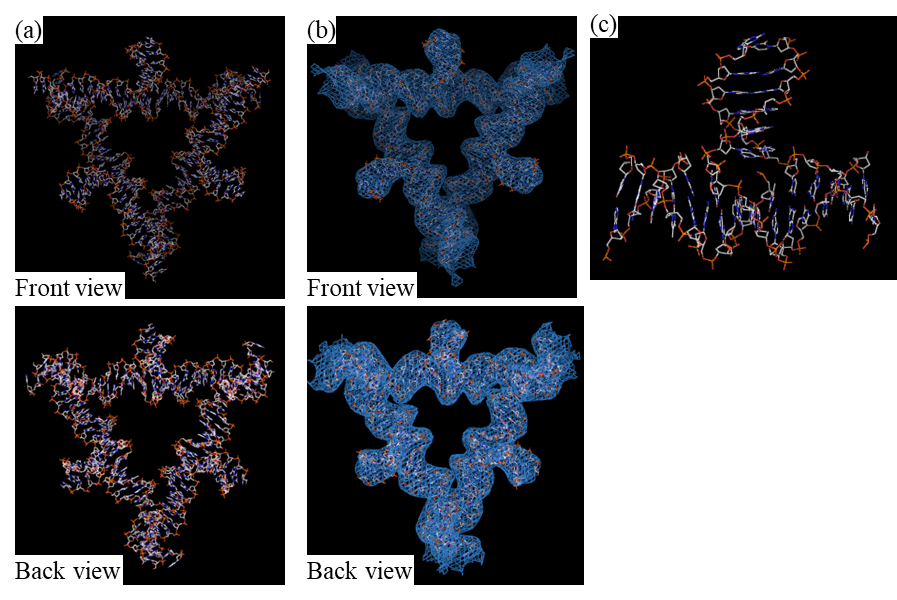


**Figure S2.** **Δ^T^7-6.** (a) structural model along the three-fold rotational axis. (b) Superimposing the final structural model and electron densities. (c) a close-up view of the T-junction.


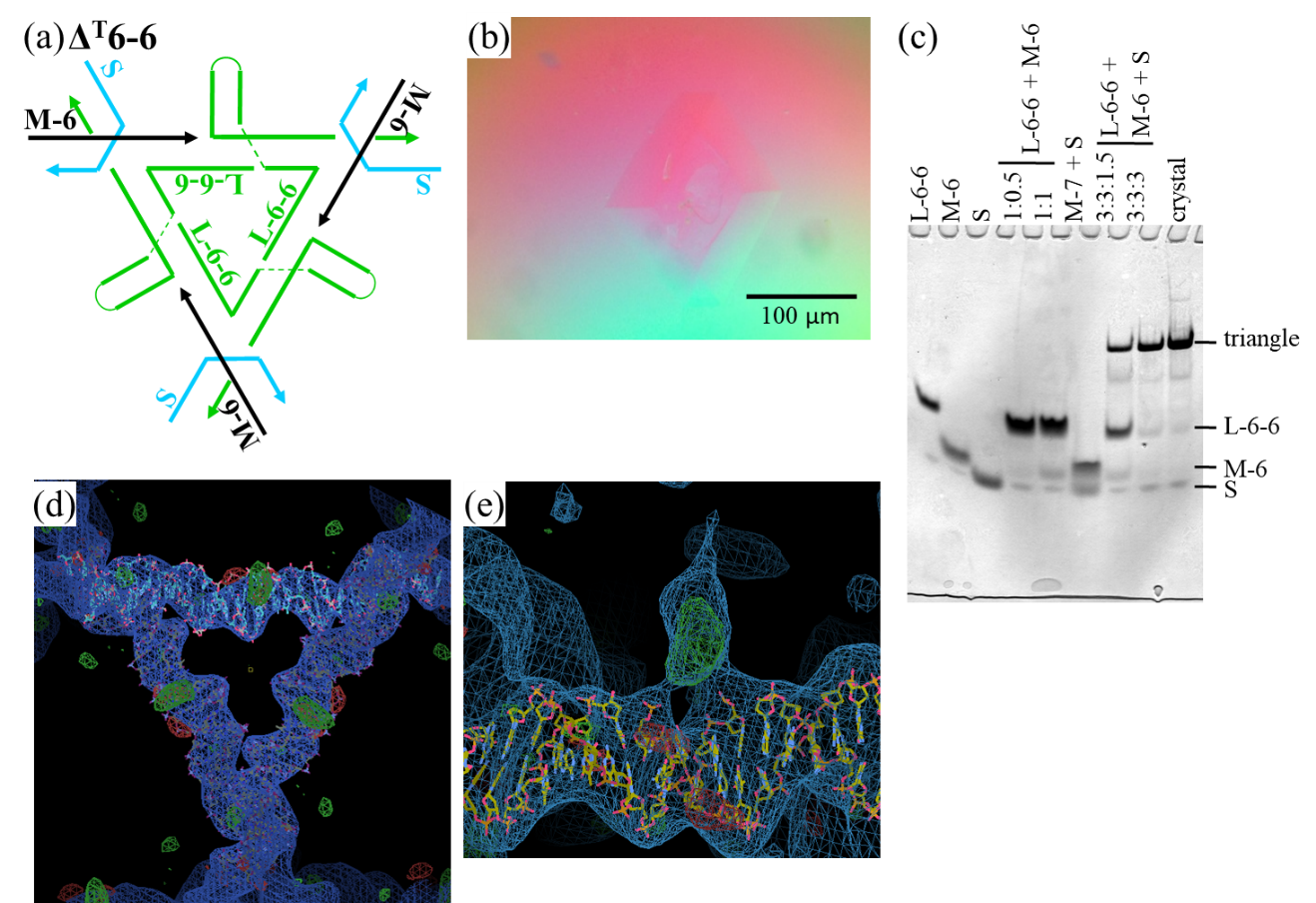

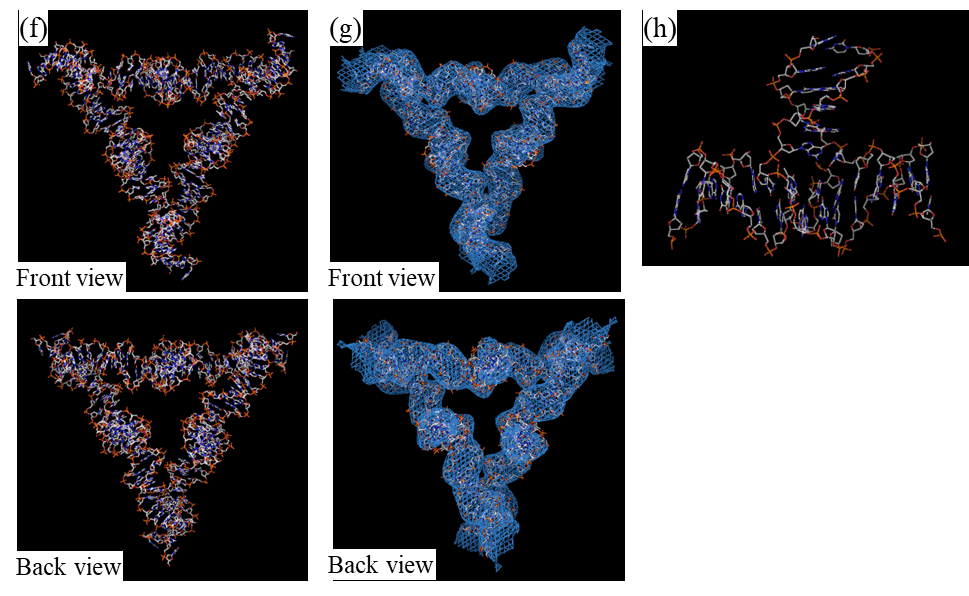


**Figure S3.** Crystallographic study of **Δ^T^6-6**. (a) Scheme of a 3-turn T-junction triangle variant. (b) An optical image of the assembled DNA crystal. (b) nPAGE (6%) analysis of the assembly of the triangle motif and crystals. The sample compositions and the chemical identify of each band are indicated above and the right side of the gel, respectively. (d) Superimposing the electron density and structure model (duplex). (e) a closed-up view of the T-junction (electron density map and duplex structural model). Note the extra electron density (green) corresponding to C-arm. (f) structural model along the three-fold rotational axis. (g) Superimposing the final structural model and electron densities. (h) a close-up view of the T-junction.


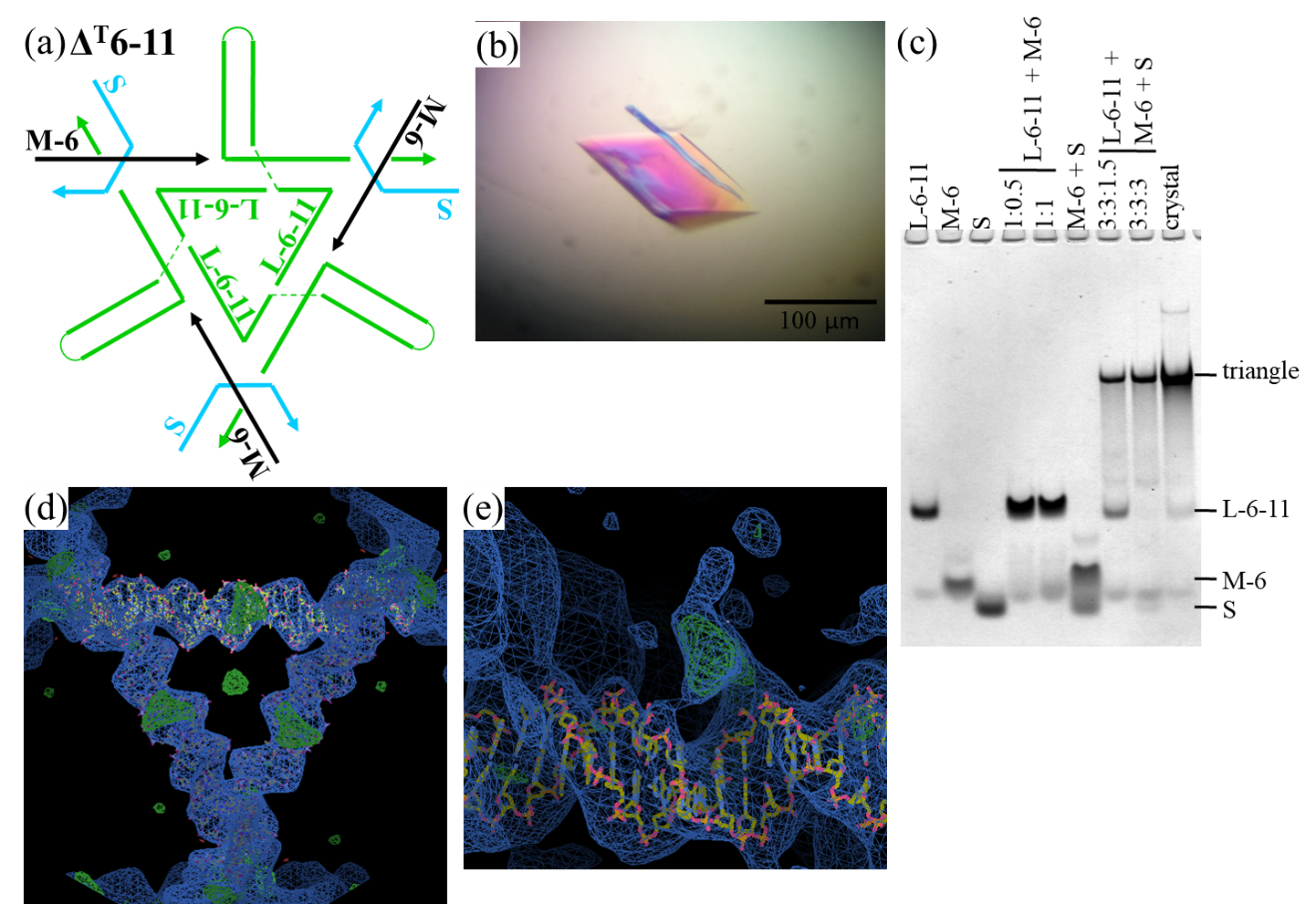

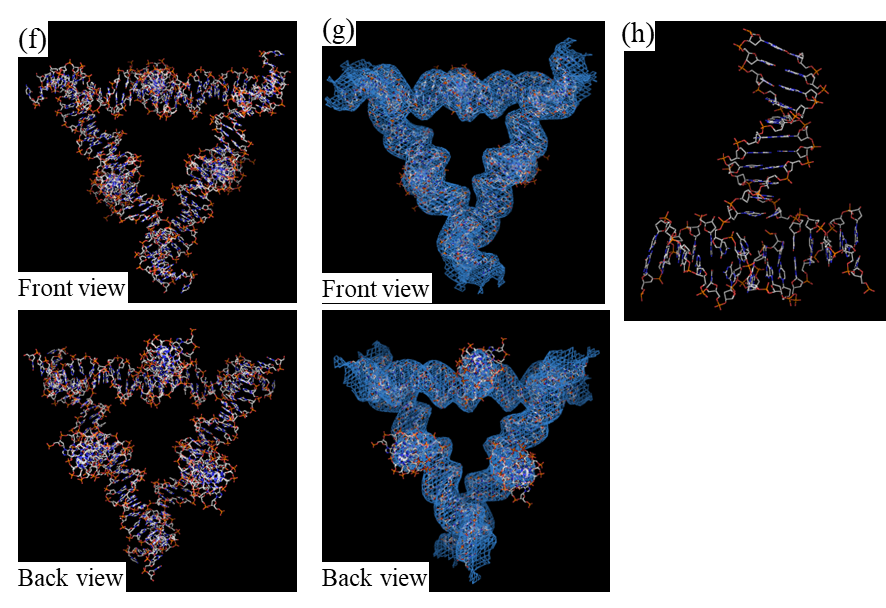


**Figure S4.** Crystallographic study of **Δ^T^6-11**. (a) Scheme of a 3-turn T-junction triangle variant. (b) An optical image of the assembled DNA crystal. (b) nPAGE (6%) analysis of the assembly of the triangle motif and crystals. The sample compositions and the chemical identify of each band are indicated above and the right side of the gel, respectively. (d) Superimposing the electron density and structure model (duplex). (e) a closed-up view of the T-junction (electron density map and duplex structural model). Note the extra electron density (green) corresponding to C-arm. (f) structural model along the three-fold rotational axis. (g) Superimposing the final structural model and electron densities. (h) a close-up view of the T-junction.


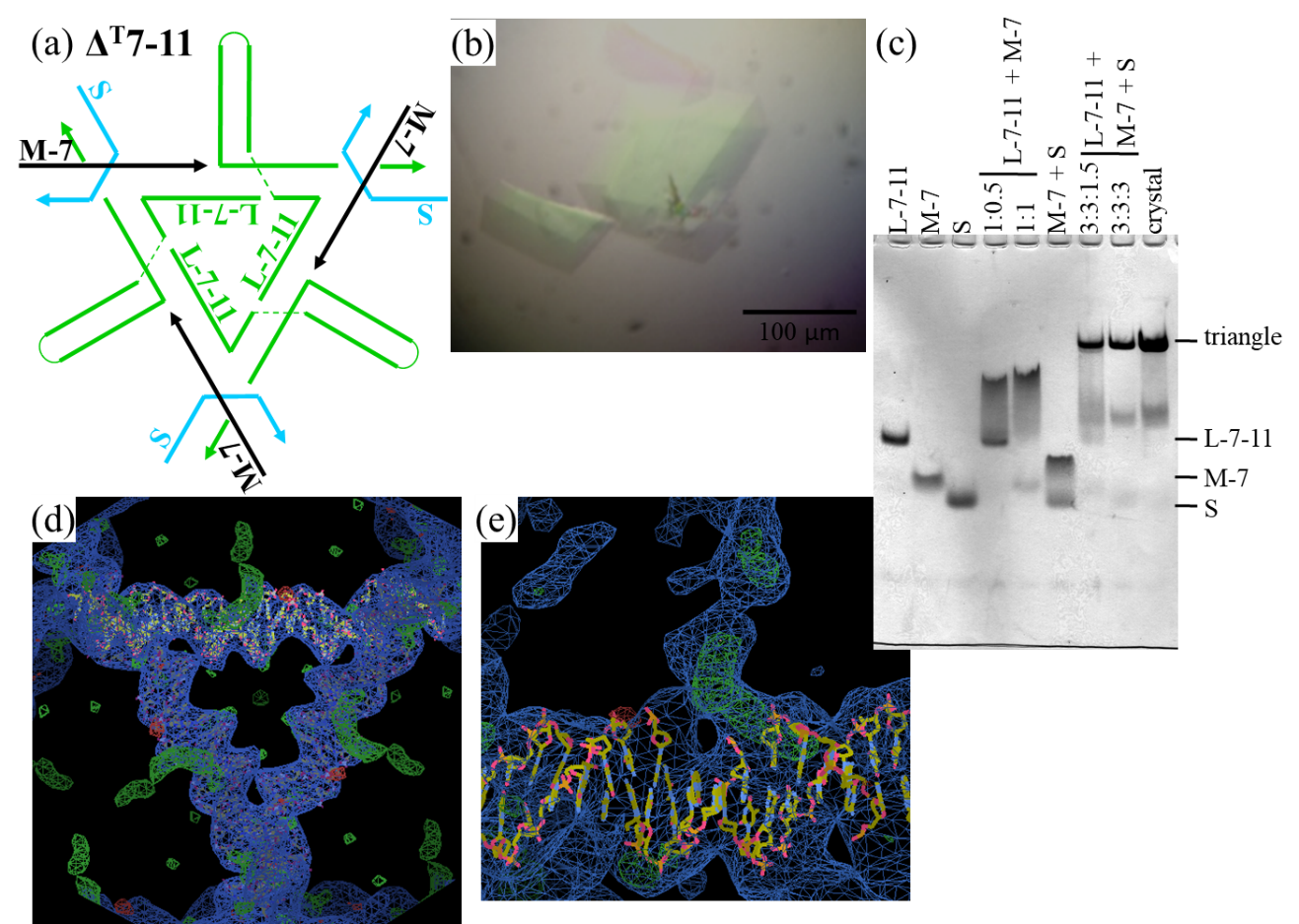

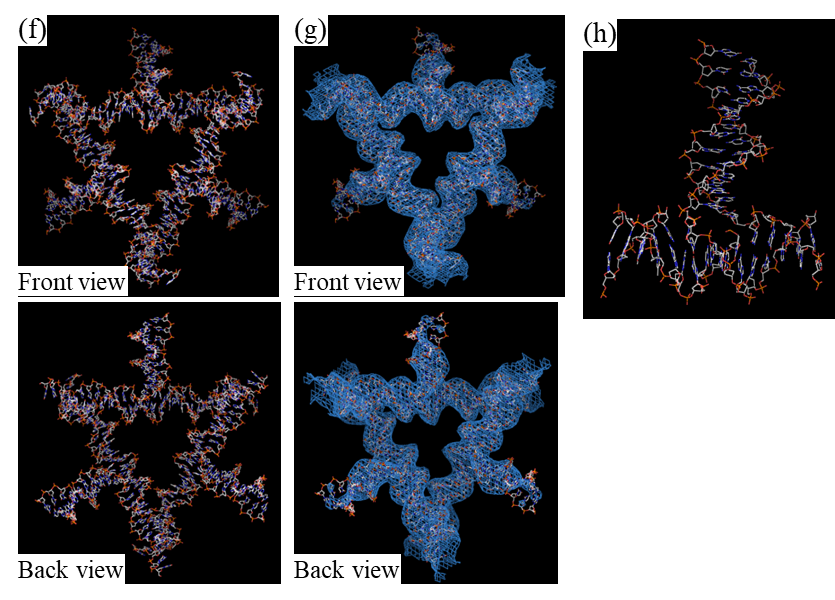


**Figure S5.** Crystallographic study of **Δ^T^7-11**. (a) Scheme of a 3-turn T-junction triangle variant. (b) An optical image of the assembled DNA crystal. (b) nPAGE (6%) analysis of the assembly of the triangle motif and crystals. The sample compositions and the chemical identify of each band are indicated above and the right side of the gel, respectively. (d) Superimposing the electron density and structure model (duplex). (e) a closed-up view of the T-junction (electron density map and duplex structural model). Note the extra electron density (green) corresponding to C-arm. (f) structural model along the three-fold rotational axis. (g) Superimposing the final structural model and electron densities. (h) a close-up view of the T-junction.


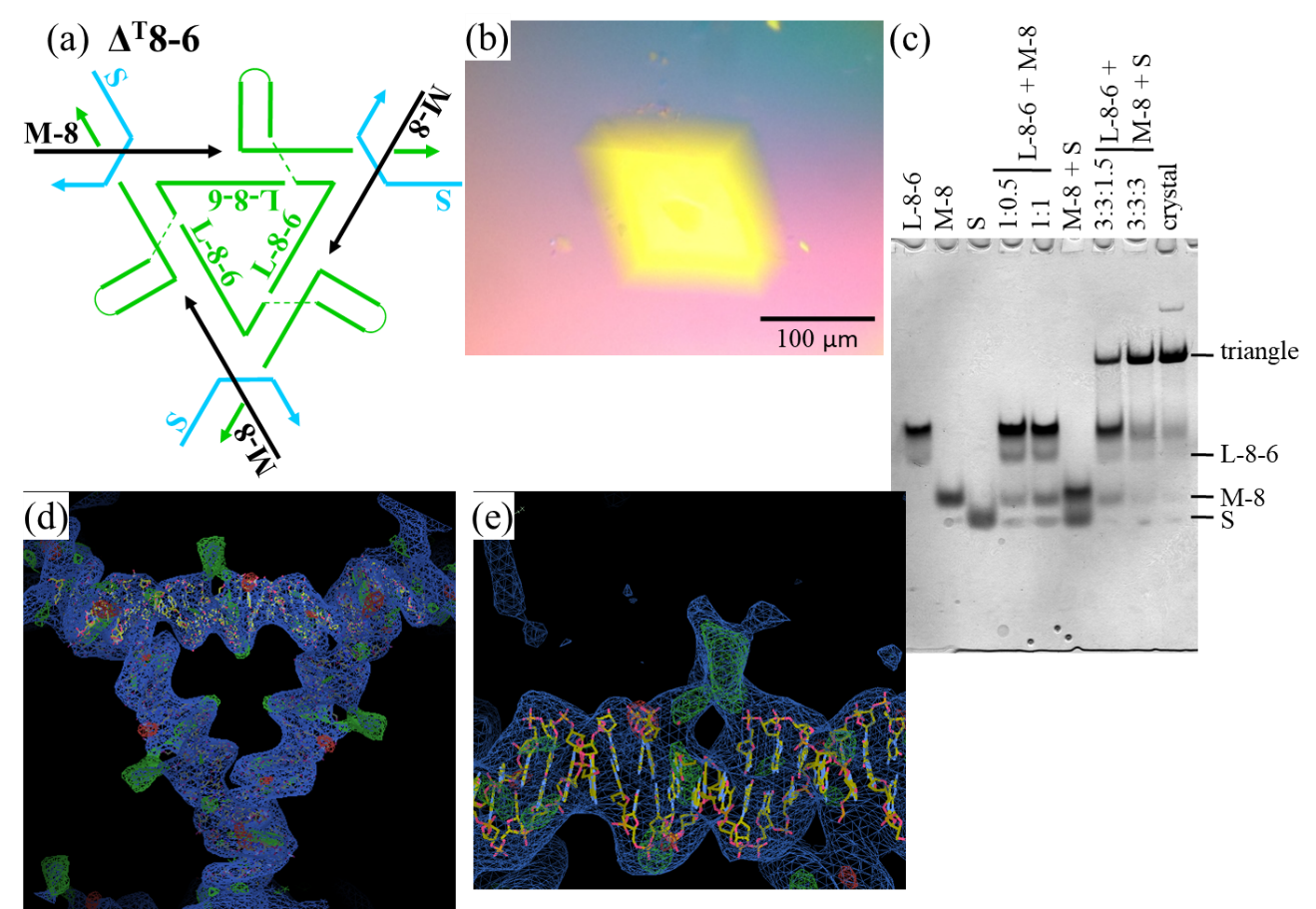

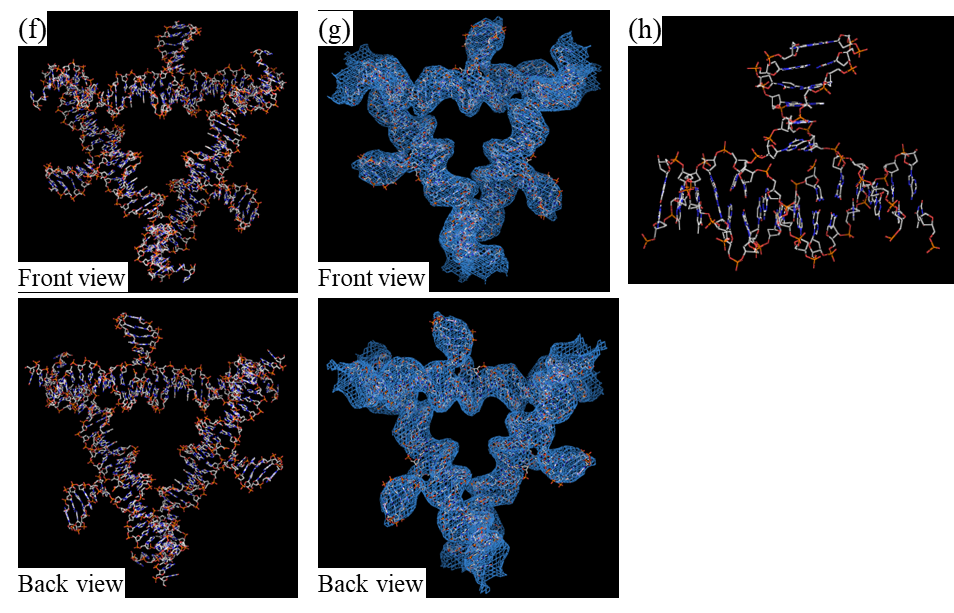


**Figure S6.** Crystallographic study of **Δ^T^8-6**. (a) Scheme of a 3-turn T-junction triangle variant. (b) An optical image of the assembled DNA crystal. (b) nPAGE (6%) analysis of the assembly of the triangle motif and crystals. The sample compositions and the chemical identify of each band are indicated above and the right side of the gel, respectively. (d) Superimposing the electron density and structure model (duplex). (e) a closed-up view of the T-junction (electron density map and duplex structural model). Note the extra electron density (green) corresponding to C-arm. (f) structural model along the three-fold rotational axis. (g) Superimposing the final structural model and electron densities. (h) a close-up view of the T-junction.


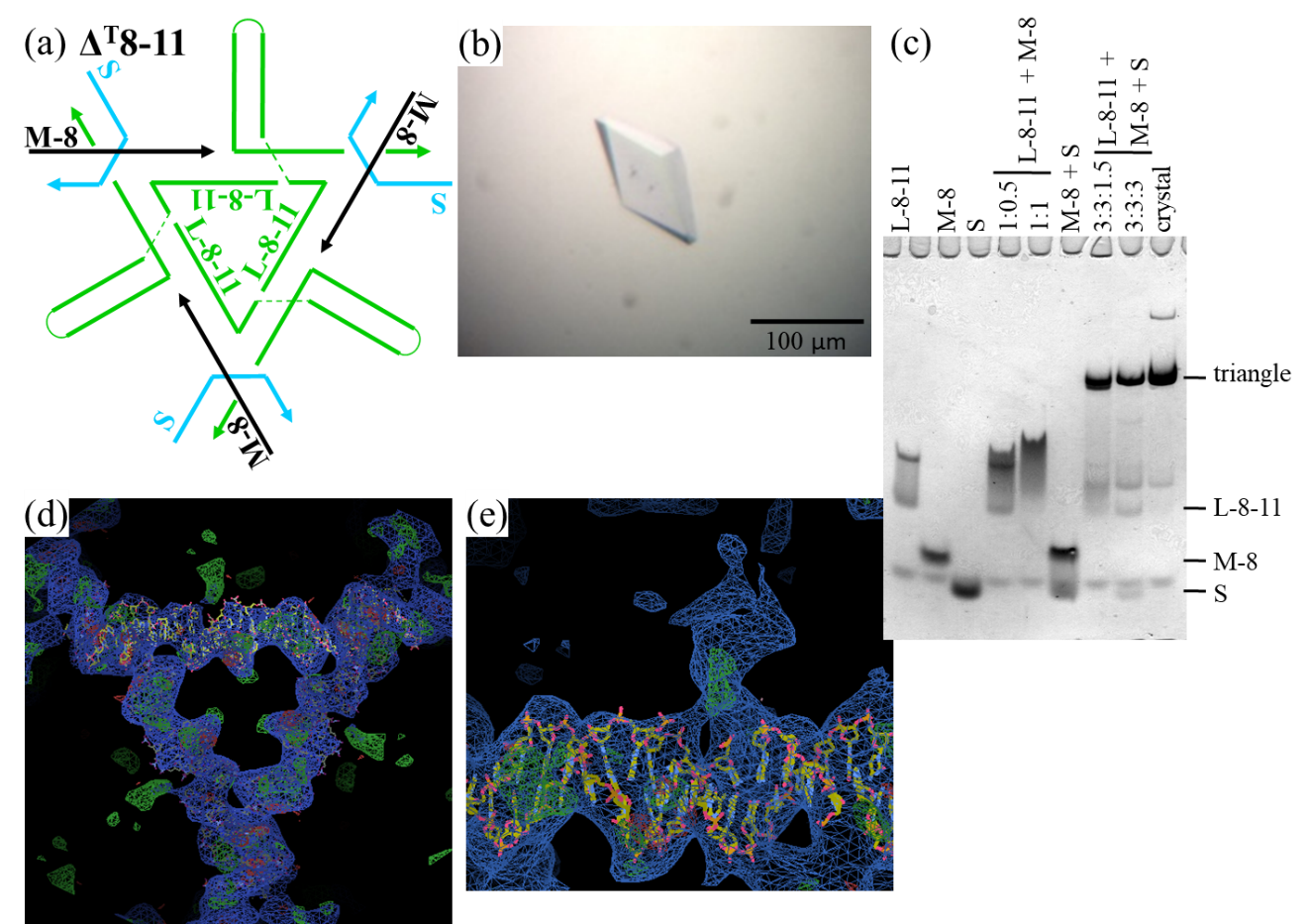

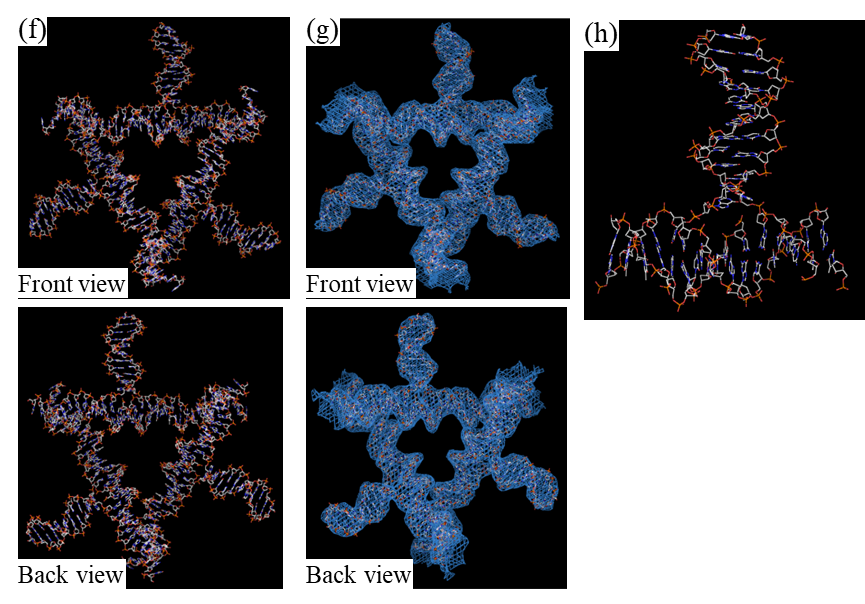


**Figure S7.** Crystallographic study of **Δ^T^8-11**. (a) Scheme of a 3-turn T-junction triangle variant. (b) An optical image of the assembled DNA crystal. (b) nPAGE (6%) analysis of the assembly of the triangle motif and crystals. The sample compositions and the chemical identify of each band are indicated above and the right side of the gel, respectively. (d) Superimposing the electron density and structure model (duplex). (e) a closed-up view of the T-junction (electron density map and duplex structural model). Note the extra electron density (green) corresponding to C-arm. (f) structural model along the three-fold rotational axis. (g) Superimposing the final structural model and electron densities. (h) a close-up view of the T-junction.


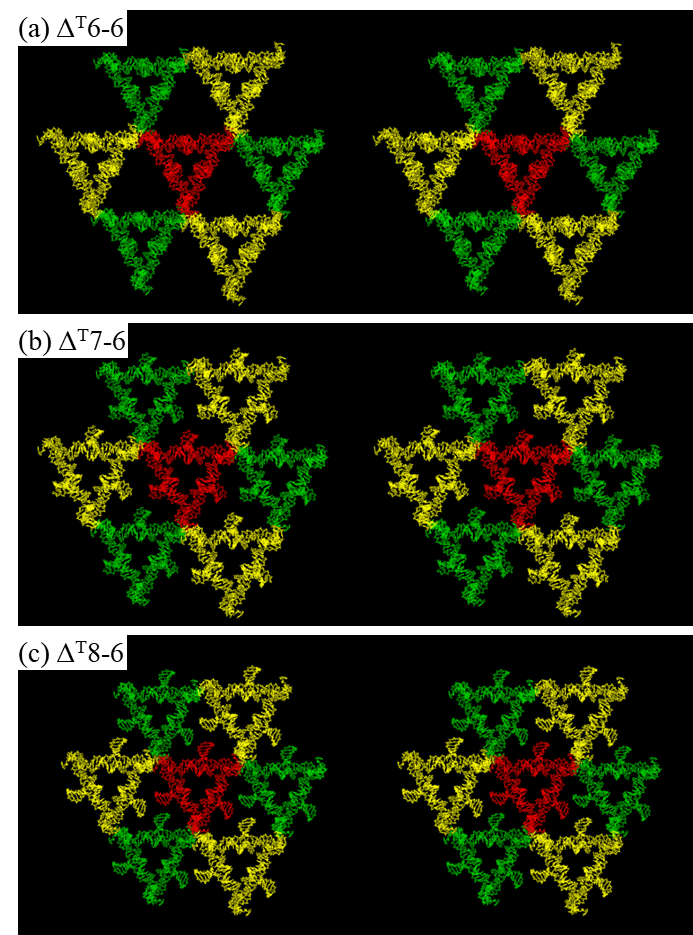

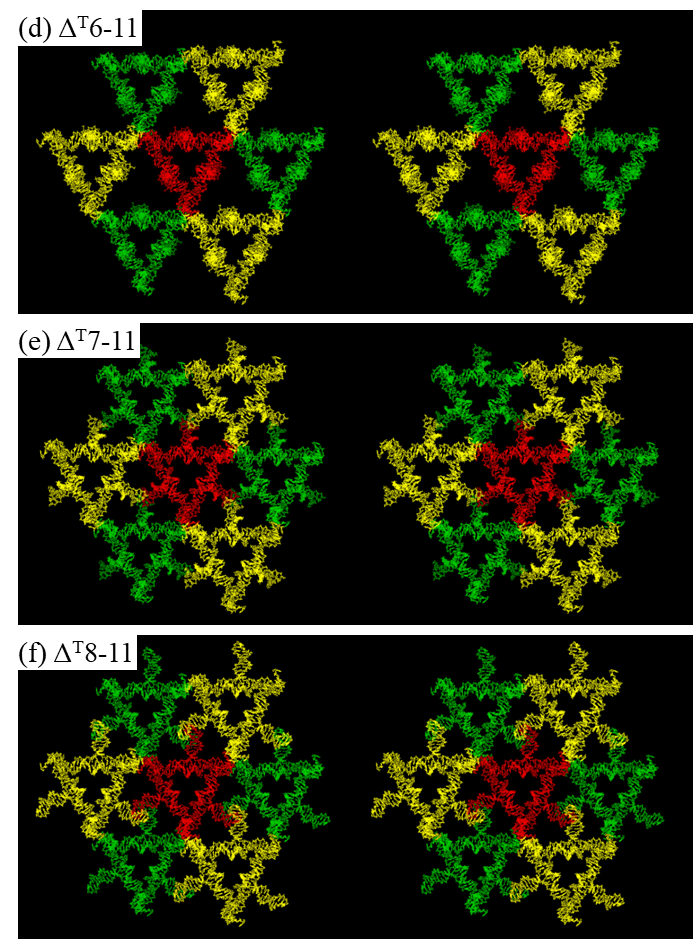


**Figure S8**. Stereo views (front) of the arrangement of T-junction incorporated triangles with neighboring motifs. (a) Δ^T^6-6, (b) Δ^T^7-6, (c) Δ^T^8-6, (d) Δ^T^6-11, (e) Δ^T^7-11, (f) Δ^T^8-11. The center red triangles connect with both yellow and green triangles through one edge. The yellow triangles locate at the plane closer to the viewer and the green triangles locate at the plane further away from the viewer. All crystals exhibited same DNA framework structures which was inherited from the original tensegrity triangle crystal.


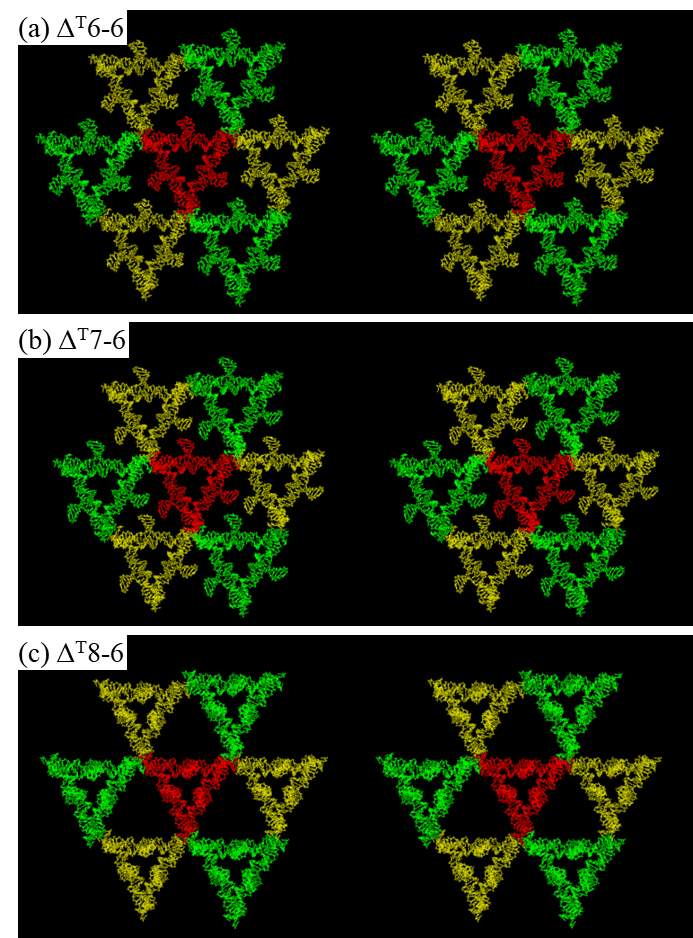

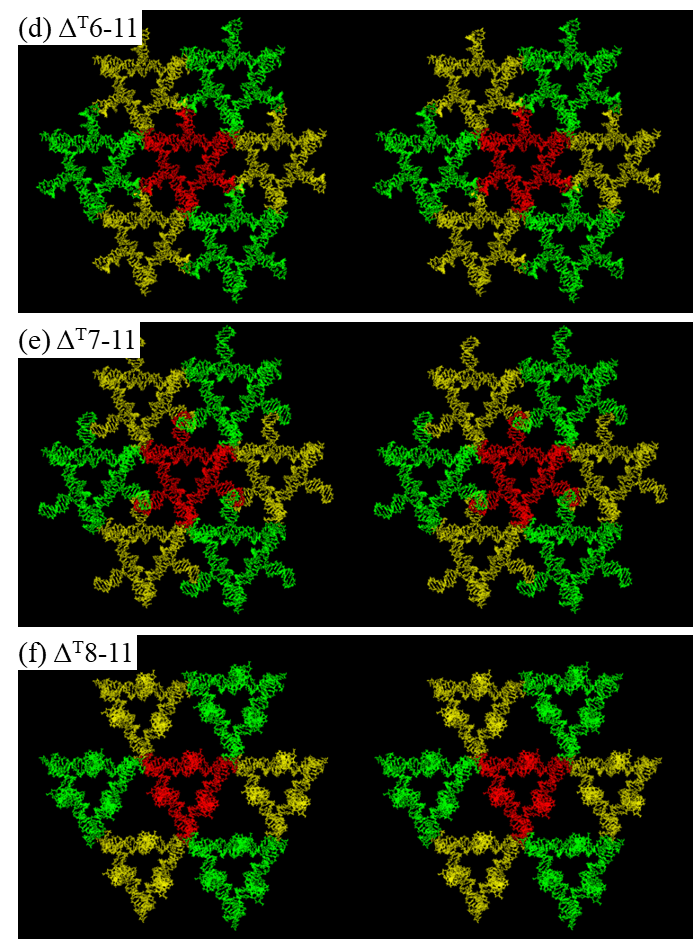


**Figure S9**. Stereo views (back) of the arrangement of T-junction incorporated triangles with neighboring motifs. (a) Δ^T^6-6, (b) Δ^T^7-6, (c) Δ^T^8-6, (d) Δ^T^6-11, (e) Δ^T^7-11, (f) Δ^T^8-11. The center red triangles connect with both yellow and green triangles through one edge. The yellow triangles locate at the plane closer to the viewer and the green triangles locate at the plane further away from the viewer. All crystals exhibited same DNA framework structures which was inherited from the original tensegrity triangle crystal.

**
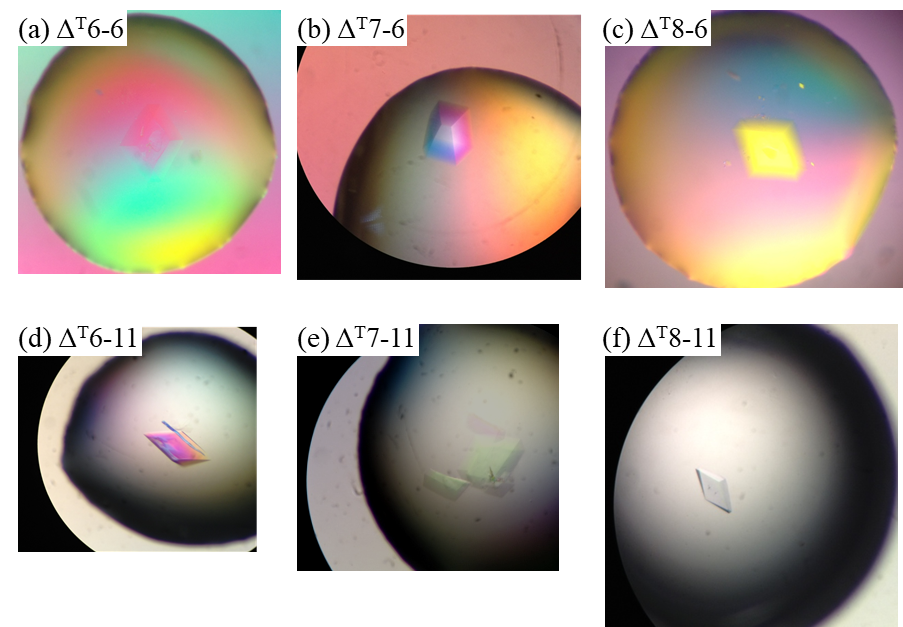
**

**Figure S10**. Optical images of examples of entire crystallization drops. In each drop, only a very few large crystals show up.

**Table S1.** Crystallography summary of all Δ^T^ variants.


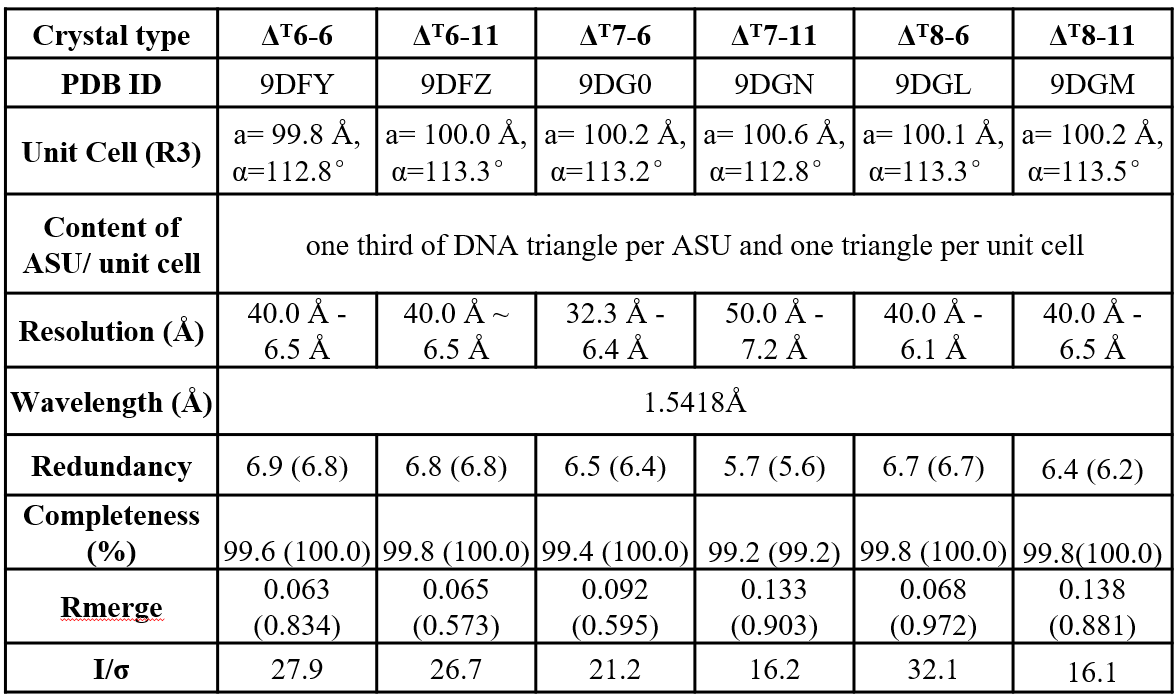


**Table S2.** Refinement statistics of all 6 Δ^T^ crystal lattices with built models.


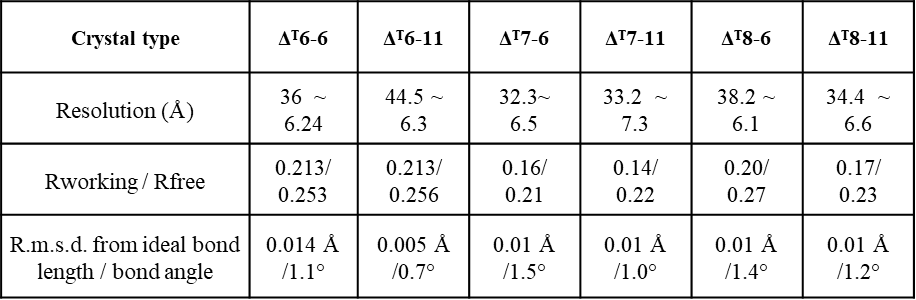

Supplement: Supplementary file 1 — Supporting Information [file ANIE-64-e18174-s002.docx]
