# Supplementary material for: Crystallographic Study of DNA T‐Junction via Crystal Engineering
Source: Angew Chem Int Ed Engl. 2025 Oct 30;64(51):e18174. doi: 10.1002/anie.202518174 (PMC12707366; doi:10.1002/anie.202518174)
Supplement: Supplementary file 2 — Supporting Information [file ANIE-64-e18174-s001.zip › renamed_966b8.pdf]

WORLDWIDE PDB PROTEIN DATA BANK

Full wwPDB X-ray Structure Validation Report (i)

Jan 30, 2025 – 02:33 AM EST

PDB ID	:	9DFY
Title	:	DNA Tensegrity Triangle with T Junctions
Deposited on	:	2024-08-30
Resolution	:	6.24 Å(reported)

This wwPDB validation report is for manuscript review

This is a Full wwPDB X-ray Structure Validation Report.

This report is produced by the wwPDB biocuration pipeline after annotation of the structure.

We welcome your comments at *validation@mail.wwpdb.org*
A user guide is available at
https://www.wwpdb.org/validation/2017/XrayValidationReportHelp
with specific help available everywhere you see the (i) symbol.

The types of validation reports are described at
http://www.wwpdb.org/validation/2017/FAQs#types.

For Manuscript Review

The following versions of software and data (see references (i)) were used in the production of this report:

MolProbity	:	4.02b-467
Xtriage (Phenix)	:	1.21
EDS	:	3.0
Percentile statistics	:	20231227.v01 (using entries in the PDB archive December 27th 2023)
CCP4	:	9.0.004 (Gargrove)
Density-Fitness	:	1.0.11
Ideal geometry (proteins)	:	Engh & Huber (2001)
Ideal geometry (DNA, RNA)	:	Parkinson et al. (1996)
Validation Pipeline (wwPDB-VP)	:	2.40

1 Overall quality at a glance (i)

The following experimental techniques were used to determine the structure:
X-RAY DIFFRACTION

The reported resolution of this entry is 6.24 Å.

Percentile scores (ranging between 0-100) for global validation metrics of the entry are shown in the following graphic. The table shows the number of entries on which the scores are based.

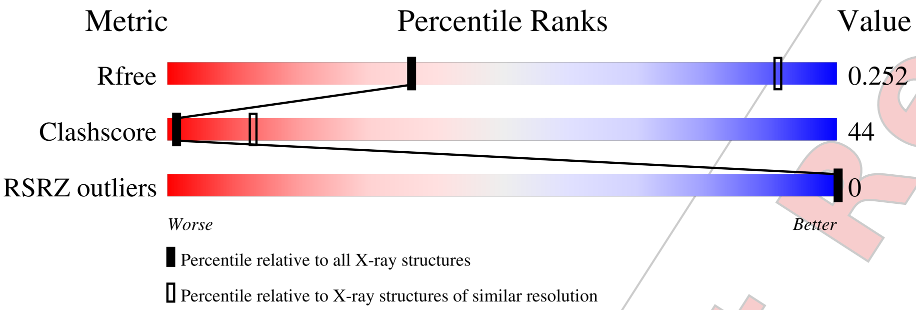

Metric	Whole archive (#Entries)	Similar resolution (#Entries, resolution range(Å))
R_{free}	164625	1088 (8.46-4.00)
Clashscore	180529	1129 (8.46-4.00)
RSRZ outliers	164620	1083 (8.46-4.00)

The table below summarises the geometric issues observed across the polymeric chains and their fit to the electron density. The red, orange, yellow and green segments of the lower bar indicate the fraction of residues that contain outliers for >=3, 2, 1 and 0 types of geometric quality criteria respectively. A grey segment represents the fraction of residues that are not modelled. The numeric value for each fraction is indicated below the corresponding segment, with a dot representing fractions <=5% The upper red bar (where present) indicates the fraction of residues that have poor fit to the electron density. The numeric value is given above the bar.

Mol	Chain	Length	Quality of chain
1	A	14	14% 71% 14%
2	B	6	17% 50% 33%
3	C	50	22% 62% 8% 8%
4	E	8	75% 25%

For Manuscript Review

2 Entry composition (i)

There are 4 unique types of molecules in this entry. The entry contains 1511 atoms, of which 0 are hydrogens and 0 are deuteriums.

In the tables below, the ZeroOcc column contains the number of atoms modelled with zero occupancy, the AltConf column contains the number of residues with at least one atom in alternate conformation and the Trace column contains the number of residues modelled with at most 2 atoms.

- Molecule 1 is a DNA chain called DNA (5'-D(*CP*AP*GP*CP*AP*GP*CP*CP*TP*GP*AP*AP*TP*A)-3').

Mol	Chain	Residues	Atoms					ZeroOcc	AltConf	Trace
			Total	C	N	O	P			
1	A	14	284	136	56	79	13	0	0	0

- Molecule 2 is a DNA chain called DNA (5'-D(P*GP*GP*CP*TP*GP*C)-3').

Mol	Chain	Residues	Atoms					ZeroOcc	AltConf	Trace
			Total	C	N	O	P			
2	B	6	124	58	23	37	6	0	0	0

- Molecule 3 is a DNA chain called DNA (50-MER).

Mol	Chain	Residues	Atoms					ZeroOcc	AltConf	Trace
			Total	C	N	O	P			
3	C	46	942	442	179	275	46	0	0	0

- Molecule 4 is a DNA chain called DNA (5'-D(*TP*GP*CP*GP*CP*TP*GP*T)-3').

Mol	Chain	Residues	Atoms					ZeroOcc	AltConf	Trace
			Total	C	N	O	P			
4	E	8	161	78	27	49	7	0	0	0

For Manuscript Review

3 Residue-property plots (i)

These plots are drawn for all protein, RNA, DNA and oligosaccharide chains in the entry. The first graphic for a chain summarises the proportions of the various outlier classes displayed in the second graphic. The second graphic shows the sequence view annotated by issues in geometry and electron density. Residues are color-coded according to the number of geometric quality criteria for which they contain at least one outlier: green = 0, yellow = 1, orange = 2 and red = 3 or more. A red dot above a residue indicates a poor fit to the electron density (RSRZ > 2). Stretches of 2 or more consecutive residues without any outlier are shown as a green connector. Residues present in the sample, but not in the model, are shown in grey.

• Molecule 1: DNA (5'-D(*CP*AP*GP*CP*AP*GP*CP*CP*TP*GP*AP*AP*TP*A)-3')

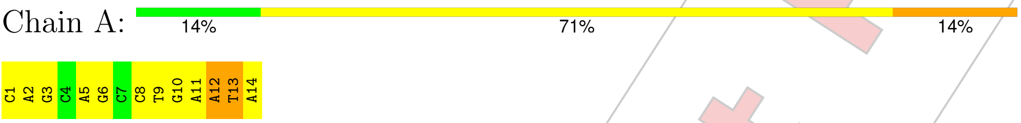

• Molecule 2: DNA (5'-D(P*GP*GP*CP*TP*GP*C)-3')

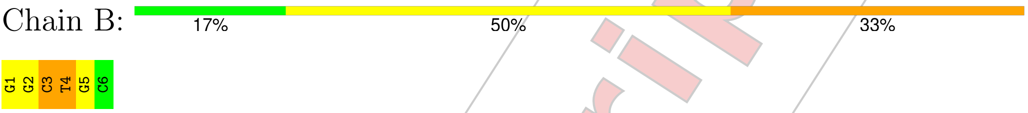

• Molecule 3: DNA (50-MER)

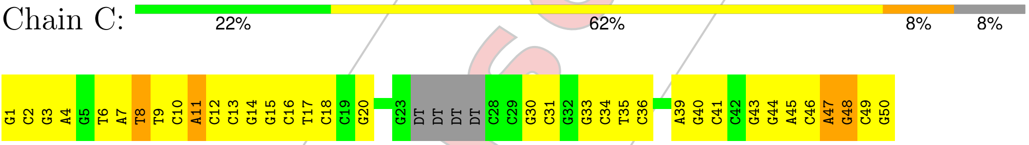

• Molecule 4: DNA (5'-D(*TP*GP*CP*GP*CP*TP*GP*T)-3')

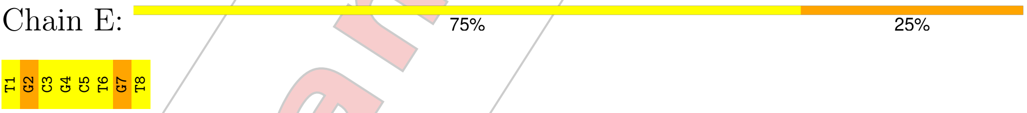

For Manuscript Review

4 Data and refinement statistics (i)

Property	Value	Source
Space group	H 3	Depositor
Cell constants<br>a, b, c, α, β, γ	166.31Å 166.31Å 82.14Å<br>90.00° 90.00° 120.00°	Depositor
Resolution (Å)	35.92 – 6.24<br>35.92 – 6.24	Depositor<br>EDS
% Data completeness<br>(in resolution range)	99.0 (35.92-6.24)<br>90.4 (35.92-6.24)	Depositor<br>EDS
R_{merge}	(Not available)	Depositor
R_{sym}	(Not available)	Depositor
$< I/\sigma(I) >$ [1]	2.07 (at 6.14Å)	Xtriage
Refinement program	PHENIX 1.20.1_4487	Depositor
R, R_{free}	0.213 , 0.253<br>0.213 , 0.252	Depositor<br>DCC
R_{free} test set	1689 reflections (10.05%)	wwPDB-VP
Wilson B-factor (Å^2)	229.1	Xtriage
Anisotropy	1.000	Xtriage
Bulk solvent k_{sol}(e/Å^3), B_{sol}(Å^2)	0.23 , 999.0	EDS
L-test for twinning[2]	$< \vert L \vert > = 0.46$, $< L^2 > = 0.29$	Xtriage
Estimated twinning fraction	0.058 for h,-h-k,-l	Xtriage
F_o,F_c correlation	0.97	EDS
Total number of atoms	1511	wwPDB-VP
Average B, all atoms (Å^2)	540.0	wwPDB-VP

Xtriage's analysis on translational NCS is as follows: *The largest off-origin peak in the Patterson function is 9.11% of the height of the origin peak. No significant pseudotranslation is detected.*

[1] Intensities estimated from amplitudes.

[2] Theoretical values of $< |L| >$, $< L^2 >$ for acentric reflections are 0.5, 0.333 respectively for untwinned datasets, and 0.375, 0.2 for perfectly twinned datasets.

For Manuscript Review

5 Model quality (i)

5.1 Standard geometry (i)

The Z score for a bond length (or angle) is the number of standard deviations the observed value is removed from the expected value. A bond length (or angle) with $|Z| > 5$ is considered an outlier worth inspection. RMSZ is the root-mean-square of all Z scores of the bond lengths (or angles).

Mol	Chain	Bond lengths		Bond angles	
		RMSZ	#$\|Z\|$ >5	RMSZ	#$\|Z\|$ >5
1	A	1.69	4/319 (1.3%)	1.31	0/490
2	B	1.69	2/138 (1.4%)	1.34	2/211 (0.9%)
3	C	1.23	2/1055 (0.2%)	1.23	5/1623 (0.3%)
4	E	1.20	0/179	1.45	2/275 (0.7%)
All	All	1.37	8/1691 (0.5%)	1.28	9/2599 (0.3%)

All (8) bond length outliers are listed below:

Mol	Chain	Res	Type	Atoms	Z	Observed(Å)	Ideal(Å)
1	A	13	DT	C1'-N1	9.51	1.61	1.49
1	A	1	DC	C1'-N1	8.86	1.60	1.49
2	B	3	DC	C1'-N1	8.37	1.60	1.49
1	A	12	DA	N9-C4	8.34	1.42	1.37
3	C	11	DA	O3'-P	6.50	1.69	1.61
1	A	12	DA	N3-C4	5.87	1.38	1.34
2	B	4	DT	N3-C4	5.56	1.43	1.38
3	C	47	DA	C6-N1	-5.24	1.31	1.35

All (9) bond angle outliers are listed below:

Mol	Chain	Res	Type	Atoms	Z	Observed(^{o})	Ideal(^{o})
3	C	11	DA	OP2-P-O3'	11.18	129.79	105.20
3	C	8	DT	N3-C4-O4	8.56	125.03	119.90
3	C	8	DT	C5-C4-O4	-8.04	119.27	124.90
3	C	11	DA	P-O3'-C3'	7.44	128.63	119.70
4	E	2	DG	O4'-C4'-C3'	-6.77	101.79	104.50
2	B	4	DT	N3-C4-O4	6.64	123.88	119.90
2	B	4	DT	C5-C4-O4	-5.95	120.73	124.90
4	E	7	DG	O4'-C1'-N9	5.33	111.73	108.00
3	C	48	DG	O4'-C1'-N9	5.29	111.70	108.00

There are no chirality outliers.

For Manuscript Review

There are no planarity outliers.

5.2 Too-close contacts (i)

In the following table, the Non-H and H(model) columns list the number of non-hydrogen atoms and hydrogen atoms in the chain respectively. The H(added) column lists the number of hydrogen atoms added and optimized by MolProbity. The Clashes column lists the number of clashes within the asymmetric unit, whereas Symm-Clashes lists symmetry-related clashes.

Mol	Chain	Non-H	H(model)	H(added)	Clashes	Symm-Clashes
1	A	284	0	158	19	1
2	B	124	0	68	12	2
3	C	942	0	513	69	2
4	E	161	0	93	27	2
All	All	1511	0	832	100	4

The all-atom clashscore is defined as the number of clashes found per 1000 atoms (including hydrogen atoms). The all-atom clashscore for this structure is 44.

All (100) close contacts within the same asymmetric unit are listed below, sorted by their clash magnitude.

Atom-1	Atom-2	Interatomic distance (Å)	Clash overlap (Å)
3:C:15:DG:H1	3:C:41:DC:N4	1.60	0.99
1:A:14:DA:N3	3:C:34:DC:C6	2.38	0.92
3:C:35:DT:O2	3:C:36:DC:N4	2.08	0.87
3:C:17:DT:H3	3:C:39:DA:H61	1.22	0.86
1:A:14:DA:C4	3:C:34:DC:C5	2.65	0.84
3:C:15:DG:N2	3:C:41:DC:N3	2.29	0.80
3:C:16:DC:N3	3:C:40:DG:O6	2.14	0.80
3:C:15:DG:H1	3:C:41:DC:H42	0.84	0.79
4:E:7:DG:C8	4:E:8:DT:H72	2.18	0.78
3:C:47:DA:H2	4:E:6:DT:H3	1.35	0.74
3:C:12:DC:C5	4:E:8:DT:H71	2.24	0.73
3:C:46:DC:N3	4:E:7:DG:O6	2.23	0.72
3:C:7:DA:H2'	3:C:8:DT:H6	1.54	0.71
3:C:16:DC:O2	3:C:40:DG:N1	2.19	0.71
1:A:14:DA:C4	3:C:34:DC:C6	2.79	0.71
3:C:20:DG:H1	3:C:31:DC:H42	1.40	0.69
3:C:12:DC:C6	4:E:8:DT:H71	2.28	0.69
3:C:3:DG:H3'	3:C:4:DA:H4'	1.75	0.69
3:C:17:DT:H3	3:C:39:DA:N6	1.92	0.67
1:A:9:DT:H2'	1:A:10:DG:C8	2.29	0.67

Continued on next page...

For Manuscript Review

Continued from previous page...

Atom-1	Atom-2	Interatomic distance (Å)	Clash overlap (Å)
3:C:13:DC:N3	3:C:43:DG:O6	2.29	0.66
3:C:12:DC:H1'	4:E:8:DT:C6	2.31	0.65
3:C:12:DC:C6	4:E:8:DT:C7	2.79	0.65
3:C:7:DA:H2'	3:C:8:DT:C6	2.31	0.64
3:C:2:DC:H2"	3:C:3:DG:N7	2.14	0.63
3:C:35:DT:H2"	3:C:36:DC:H5	1.63	0.62
2:B:1:DG:H3'	2:B:2:DG:C8	2.35	0.61
3:C:47:DA:N1	4:E:6:DT:O4	2.35	0.60
1:A:14:DA:H1'	3:C:34:DC:H6	1.68	0.59
3:C:46:DC:O2	4:E:7:DG:N1	2.27	0.58
1:A:5:DA:H61	2:B:4:DT:H3	1.52	0.56
2:B:2:DG:H2'	2:B:3:DC:O4'	2.06	0.55
3:C:44:DG:H2"	3:C:45:DA:H8	1.70	0.55
3:C:12:DC:C2	4:E:8:DT:C4	2.94	0.55
1:A:11:DA:N3	1:A:12:DA:N7	2.55	0.54
4:E:2:DG:C2	4:E:3:DC:N4	2.76	0.54
3:C:3:DG:C3'	3:C:4:DA:H4'	2.37	0.53
1:A:9:DT:H2'	1:A:10:DG:H8	1.73	0.53
4:E:2:DG:H2'	4:E:3:DC:C6	2.44	0.52
3:C:50:DG:C6	4:E:3:DC:N3	2.77	0.52
1:A:13:DT:H4'	1:A:14:DA:OP1	2.09	0.51
3:C:20:DG:H1	3:C:31:DC:N4	2.08	0.51
3:C:14:DG:C4	3:C:43:DG:N2	2.78	0.51
3:C:14:DG:H2'	3:C:15:DG:H4'	1.91	0.51
4:E:4:DG:C2	4:E:5:DC:C2	2.99	0.51
3:C:15:DG:H2'	3:C:16:DC:C4	2.46	0.51
1:A:5:DA:H8	1:A:5:DA:H5'	1.76	0.50
3:C:12:DC:H1'	4:E:8:DT:C5	2.47	0.50
3:C:50:DG:O6	4:E:3:DC:N3	2.44	0.50
3:C:30:DG:C2	3:C:31:DC:C2	3.00	0.49
1:A:14:DA:H1'	3:C:34:DC:C6	2.46	0.49
4:E:4:DG:N2	4:E:5:DC:O4'	2.46	0.49
1:A:10:DG:H2"	1:A:11:DA:O4'	2.13	0.49
3:C:47:DA:C2	3:C:48:DG:C2	3.01	0.49
3:C:33:DG:H2"	3:C:35:DT:H73	1.95	0.49
1:A:8:DC:O2	2:B:2:DG:C2	2.66	0.49
3:C:14:DG:H21	3:C:16:DC:H5'	1.78	0.48
3:C:6:DT:H2"	3:C:7:DA:N7	2.28	0.48
4:E:7:DG:N9	4:E:8:DT:H72	2.29	0.48
1:A:2:DA:C4	1:A:3:DG:N7	2.82	0.47
3:C:43:DG:H2"	3:C:44:DG:C8	2.48	0.47

Continued on next page...

For Manuscript Review

Continued from previous page...

Atom-1	Atom-2	Interatomic distance (Å)	Clash overlap (Å)
2:B:1:DG:N2	2:B:2:DG:C5	2.83	0.47
3:C:1:DG:H2'	3:C:1:DG:N3	2.30	0.47
3:C:12:DC:C6	4:E:8:DT:C5	3.03	0.47
3:C:44:DG:N3	3:C:45:DA:C8	2.83	0.46
3:C:46:DC:H2"	3:C:47:DA:C8	2.51	0.46
3:C:50:DG:N1	4:E:3:DC:O2	2.47	0.45
1:A:5:DA:C6	2:B:5:DG:C2	3.05	0.44
1:A:5:DA:N6	2:B:4:DT:H3	2.16	0.44
3:C:10:DC:N4	3:C:11:DA:N6	2.66	0.44
3:C:47:DA:H1'	3:C:48:DG:C8	2.52	0.44
4:E:6:DT:H2"	4:E:7:DG:C8	2.52	0.44
3:C:9:DT:C2	3:C:10:DC:C4	3.06	0.44
3:C:12:DC:N1	4:E:8:DT:C4	2.86	0.44
3:C:47:DA:H1'	3:C:48:DG:C4	2.53	0.44
2:B:2:DG:C2	2:B:3:DC:H1'	2.53	0.43
3:C:46:DC:H2"	3:C:47:DA:N7	2.33	0.43
1:A:5:DA:H5'	1:A:5:DA:C8	2.54	0.42
1:A:13:DT:H2"	1:A:14:DA:C8	2.54	0.42
2:B:1:DG:H3'	2:B:2:DG:H8	1.84	0.42
3:C:18:DC:N4	3:C:33:DG:H1	2.18	0.42
3:C:34:DC:C5	3:C:35:DT:H72	2.54	0.42
3:C:9:DT:O2	3:C:10:DC:C4	2.73	0.42
3:C:44:DG:C2	3:C:45:DA:C5	3.08	0.42
3:C:14:DG:C5	3:C:43:DG:N2	2.88	0.42
3:C:49:DC:H2'	3:C:50:DG:H8	1.84	0.42
3:C:17:DT:O2	3:C:39:DA:N1	2.52	0.42
3:C:2:DC:H4'	3:C:3:DG:OP1	2.20	0.42
3:C:16:DC:N3	3:C:40:DG:C6	2.87	0.41
1:A:5:DA:H2"	1:A:6:DG:O4'	2.20	0.41
2:B:1:DG:N3	2:B:1:DG:H2'	2.35	0.41
3:C:12:DC:H1'	4:E:8:DT:N1	2.34	0.41
3:C:12:DC:C2	4:E:8:DT:O4	2.74	0.41
3:C:14:DG:C3'	3:C:15:DG:H4'	2.51	0.41
3:C:46:DC:N3	4:E:7:DG:C6	2.89	0.41
3:C:47:DA:H2"	3:C:48:DG:C8	2.55	0.41
4:E:4:DG:H2"	4:E:5:DC:H5'	2.02	0.41
2:B:2:DG:C2	2:B:3:DC:O2	2.74	0.41
4:E:1:DT:O2	4:E:2:DG:H1'	2.21	0.41
2:B:2:DG:N1	2:B:3:DC:O2	2.54	0.40

All (4) symmetry-related close contacts are listed below. The label for Atom-2 includes the symmetry operator and encoded unit-cell translations to be applied.

For Manuscript Review

Atom-1	Atom-2	Interatomic distance (Å)	Clash overlap (Å)
2:B:1:DG:OP1	4:E:8:DT:O3'[2_555]	1.25	0.95
2:B:1:DG:P	4:E:8:DT:O3'[2_555]	1.41	0.79
1:A:12:DA:N6	3:C:8:DT:N3[2_555]	2.14	0.06
3:C:4:DA:N6	3:C:35:DT:O4[3_555]	2.15	0.05

5.3 Torsion angles (i)

5.3.1 Protein backbone (i)

There are no protein molecules in this entry.

5.3.2 Protein sidechains (i)

There are no protein molecules in this entry.

5.3.3 RNA (i)

There are no RNA molecules in this entry.

5.4 Non-standard residues in protein, DNA, RNA chains (i)

There are no non-standard protein/DNA/RNA residues in this entry.

5.5 Carbohydrates (i)

There are no oligosaccharides in this entry.

5.6 Ligand geometry (i)

There are no ligands in this entry.

5.7 Other polymers (i)

There are no such residues in this entry.

For Manuscript Review

5.8 Polymer linkage issues (i)

There are no chain breaks in this entry.

For Manuscript Review

6 Fit of model and data (i)

6.1 Protein, DNA and RNA chains (i)

In the following table, the column labelled '#RSRZ> 2' contains the number (and percentage) of RSRZ outliers, followed by percent RSRZ outliers for the chain as percentile scores relative to all X-ray entries and entries of similar resolution. The OWAB column contains the minimum, median, 95th percentile and maximum values of the occupancy-weighted average B-factor per residue. The column labelled 'Q< 0.9' lists the number of (and percentage) of residues with an average occupancy less than 0.9.

Mol	Chain	Analysed	<RSRZ>	#RSRZ>2			OWAB(Å^2)	Q<0.9
1	A	14/14 (100%)	0.26	0	100	100	284, 373, 554, 567	0
2	B	6/6 (100%)	0.04	0	100	100	361, 401, 475, 522	0
3	C	46/50 (92%)	0.47	0	100	100	348, 585, 798, 820	0
4	E	8/8 (100%)	-0.12	0	100	100	251, 380, 485, 568	0
All	All	74/78 (94%)	0.33	0	100	100	251, 516, 796, 820	0

There are no RSRZ outliers to report.

6.2 Non-standard residues in protein, DNA, RNA chains (i)

There are no non-standard protein/DNA/RNA residues in this entry.

6.3 Carbohydrates (i)

There are no monosaccharides in this entry.

6.4 Ligands (i)

There are no ligands in this entry.

6.5 Other polymers (i)

There are no such residues in this entry.

For Manuscript Review
